# Genome‐wide analysis of epigenetic and transcriptional changes associated with heterosis in pigeonpea

**DOI:** 10.1111/pbi.13333

**Published:** 2020-02-03

**Authors:** Pallavi Sinha, Vikas K. Singh, Rachit K. Saxena, Sandip M. Kale, Yuqi Li, Vanika Garg, Tang Meifang, Aamir W. Khan, Kyung Do Kim, Annapurna Chitikineni, K. B. Saxena, C. V. Sameer Kumar, Xin Liu, Xun Xu, Scott Jackson, Wayne Powell, Eviatar Nevo, Iain R. Searle, Mukesh Lodha, Rajeev K. Varshney

**Affiliations:** ^1^ International Crops Research Institute for the Semi‐Arid Tropics (ICRISAT) Patancheru India; ^2^ International Rice Research Institute, South‐Asia Hub Patancheru India; ^3^ The Leibniz Institute of Plant Genetics and Crop Plant Research Gatersleben Germany; ^4^ BGI‐Shenzhen Shenzhen China; ^5^ University of Georgia Athens USA; ^6^ Myongji University Yongin Republic of Korea; ^7^ Scotland's Rural College (SRUC) Edinburgh UK; ^8^ University of Haifa Haifa Israel; ^9^ The University of Adelaide Adelaide Australia; ^10^ Centre for Cellular and Molecular Biology (CSIR) Hyderabad India

**Keywords:** epigenomics, differentially expression gene, heterosis, pigeonpea, small RNA, miRNA

## Abstract

Hybrids are extensively used in agriculture to deliver an increase in yield, yet the molecular basis of heterosis is not well understood. Global DNA methylation analysis, transcriptome analysis and small RNA profiling were aimed to understand the epigenetic effect of the changes in gene expression level in the two hybrids and their parental lines. Increased DNA methylation was observed in both the hybrids as compared to their parents. This increased DNA methylation in hybrids showed that majority of the 24‐nt siRNA clusters had higher expression in hybrids than the parents. Transcriptome analysis revealed that various phytohormones (auxin and salicylic acid) responsive hybrid‐MPV DEGs were significantly altered in both the hybrids in comparison to MPV. DEGs associated with plant immunity and growth were overexpressed whereas DEGs associated with basal defence level were repressed. This antagonistic patterns of gene expression might contribute to the greater growth of the hybrids. It was also noticed that some common as well as unique changes in the regulatory pathways were associated with heterotic growth in both the hybrids. Approximately 70% and 67% of down‐regulated hybrid‐MPV DEGs were found to be differentially methylated in ICPH 2671 and ICPH 2740 hybrid, respectively. This reflected the association of epigenetic regulation in altered gene expressions. Our findings also revealed that miRNAs might play important roles in hybrid vigour in both the hybrids by regulating their target genes, especially in controlling plant growth and development, defence and stress response pathways. The above finding provides an insight into the molecular mechanism of pigeonpea heterosis.

## Introduction

Pigeonpea is the sixth most important legume crop, cultivated on ~7.03 million ha (m ha), with a production of ~4.89 million tons (mt), globally (FAO, 2017). Importantly, it is an important protein source in parts of Asia and a cash crop to millions of resource‐poor farmers in Asia and Africa (Mulla and Saxena, 2010). Yield stagnation in pigeonpea has been a major concern and remains a challenge; although, cytoplasmic male sterility (CMS)‐based hybrid system has been developed in pigeonpea to increase yields by exploiting heterosis (Saxena *et al.*, 2005). The first commercial food legume pigeonpea hybrid, ICPH 2671, was released for cultivation (Saxena *et al.*, 2013) followed by ICPH 3762 (Saxena and Tikle, 2015) and ICPH 2740 (Saxena, 2015). These hybrids have >30% higher yield over the local varieties in farmers' fields showing that higher yields in pigeonpea can be achieved.

Hybrid vigour or heterosis refers to the superior performance of F_1_ hybrid plants over their parents exploited well in several commercial crop breeding programmes. However, the underlying molecular mechanisms involved to explain heterosis remain largely unknown (Govindaraju, 2019). Classical genetics explanations include dominance, overdominance and epistasis hypotheses have each been proposed; however, these hypotheses are not well connected to the genome‐level data and do not explain the molecular basis of heterosis. The potential molecular mechanism of heterosis is associated with genomic and epigenetic modification in hybrids. These modifications, in turn, give advantages in growth, stress resistance and adaptability in F_1_ hybrids over their parents because of interactions between alleles of parental genomes that change the regulatory network of related genes. High‐throughput sequencing technologies have enabled detailed investigations of the molecular basis of heterosis at the whole genome level (Groszmann *et al.*, 2011; Ni *et al.*, 2009; Song *et al.*, 2010).

The role of epigenetics variation in heterosis and association of small RNA with DNA methylation has been reported in many crops (Chen, 2013; Greaves *et al.*, 2015). Dapp *et al. *(2015) demonstrated the contribution of epigenetic regulation in heterosis and their extent by using epiRILs with varying levels and distribution of DNA methylation in Arabidopsis. The regions with non‐additive changes in the DNA methylation levels at loci where parental methylation levels are different are known as differentially methylated regions (DMRs) (Zhang *et al.*, 2016). These DMRs attributed by two mechanisms, transchromosomal methylation (TCM) and transchromosomal demethylation (TCdM), whereby the methylation level of one parental allele is altered to resemble the methylation level of the other parental allele (Greaves *et al.*, 2012). TCM and TCdM refer to allelic interactions of specific chromosomal loci. Several studies investigated the global patterns of natural variation in epigenetic modifications and small RNAs, and their relationships with transcriptomic polymorphisms (Lewsey *et al.*, 2016; Shen *et al.*, 2012). These epigenetic modifications cause changes in biological pathways and phenotypic traits in hybrids, which include energy, metabolism and biomass, light and hormonal signalling, stress responses and ageing, and flowering, fruiting and yield (Chen, 2013). Similarly, it was found that locus‐specific epigenetic divergence between the parental lines can directly or indirectly trigger heterosis in Arabidopsis hybrids, independent of genetic changes (Lauss *et al.*, 2018). Altered phytohormones‐related pathways were also found in many studies controlling heterosis related genes in hybrids (Shen *et al.*, 2017). Similarly, several efforts were made to understand the molecular basis of F_1_ heterosis in the case of commercial crops like rice (He *et al.*, 2010), maize (He *et al.*, 2013) and brassica (Shen *et al.*, 2017).

Small RNAs, including small interfering RNAs (siRNAs) and microRNAs (miRNAs), regulate gene expression through epigenetic modifications and by posttranscriptional mechanisms (Lu *et al.*, 2006; Vaucheret, 2006). Despite the difference in the origin and generation of their precursors, both siRNAs and miRNAs require DICER proteins for processing, and both are assembled into the RNA‐induced silencing complex (RISC) to target their complementary RNAs (Bartel, 2004). The siRNAs regulate gene expression by directing DNA methylation, particularly in transposable elements (TEs) and a very small number of protein‐coding genes. However, cis‐ and trans‐regulating miRNAs influence natural variation in several metabolic pathways that affect growth vigour and stress responses (Lewsey *et al.*, 2016; Ng *et al.*, 2012).

Although pigeonpea used to be considered an orphan crop, development of large‐scale genomic resources such as genome assembly (Varshney *et al.*, 2012), genome re‐sequencing (Varshney *et al.*, 2017), several genetic maps (Saxena *et al.*, 2019; Saxena *et al.*, 2018; Saxena *et al.*, 2017), gene expression atlas (Pazhamala *et al.*, 2017), have put the pigeonpea crop together with major/extensively studied crops that are rich in genomic resources. The completion of a draft genome sequence of pigeonpea showed a quantum jump in its status and joined the league of model/genomic resource‐rich crops (Varshney *et al.*, 2019). Genome assembly of pigeonpea represents assembly of 72.7% (605.78 Mb) of the 833.07 Mb pigeonpea genome (Varshney *et al.*, 2012). With the availability of high‐throughput sequencing technologies, recently 292 accessions from the reference set, including 117 breeding lines, 166 landraces, 2 others and 7 accessions from three wild species were sequenced and genomic regions associated with domestication and agronomic traits were identified (Varshney *et al.*, 2017).

In this context, the current study aims to design a comprehensive analysis of bisulfite sequencing, transcriptome sequencing and small RNA profiling of two hybrids and their parental lines. We found that both hybrids had increased DNA methylation throughout the entire genome, predominantly in the regions associated with sRNAs. We observed changes in phytohormones (auxin and salicylic acid) regulated plant growth defence and stress‐responsive genes in both the hybrids and compared with mid‐parent value (MPV). Genes associated with plant growth and stress response were up‐regulated whereas, genes associated with defence were down‐regulated in both the hybrids. Further, epigenetic modifications (DMGs: methylated‐hybrid‐MPV DEGs) in the key genes associated with the identified regulatory pathways associated with heterosis were also observed. We also analysed the role of miRNAs and their interaction with their target genes in heterosis. Overall, we report that DNA methylation may play a potential role in heterosis and genome‐wide re‐modelling of gene expression in hybrids is expected to provide an opportunity to understand and exploit this complex trait for crop improvement programmes.

## Results

**Figure 1:**
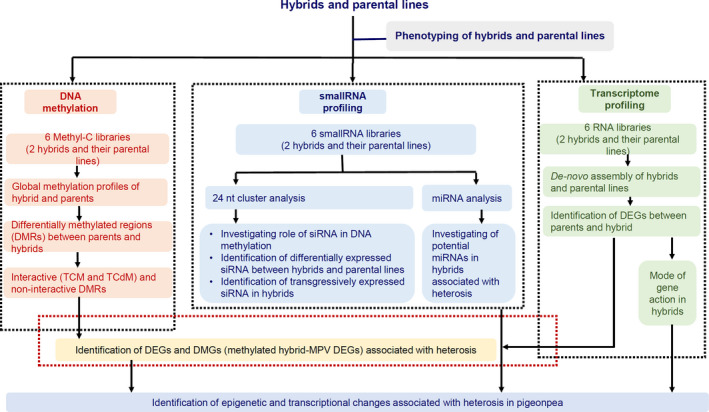
The overall workflow conducted to investigate the heterosis mechanism in pigeonpea. Two hybrids and their parental lines were selected for this analysis. Firstly, parental lines and hybrids were characterized phenotypically in 15 and 30 days after sowing. The 15‐days seedling was utilized to prepare the libraries for DNA methylation, small RNA and RNA sequencing. Several bioinformatics pipelines were utilized to decipher the genome‐wide data to answers the biological questions. As mentioned in the figure, we have performed several different analyses of the data sets to understand the heterosis in pigeonpea.

To get a better understanding of heterosis in pigeonpea hybrids, we examined the methylomes, transcriptome and small RNA of two leading pigeonpea hybrids and their parental lines (Figure 1).

### Hybrids show significant heterosis over parents at the seedling stage

**Figure 2:**
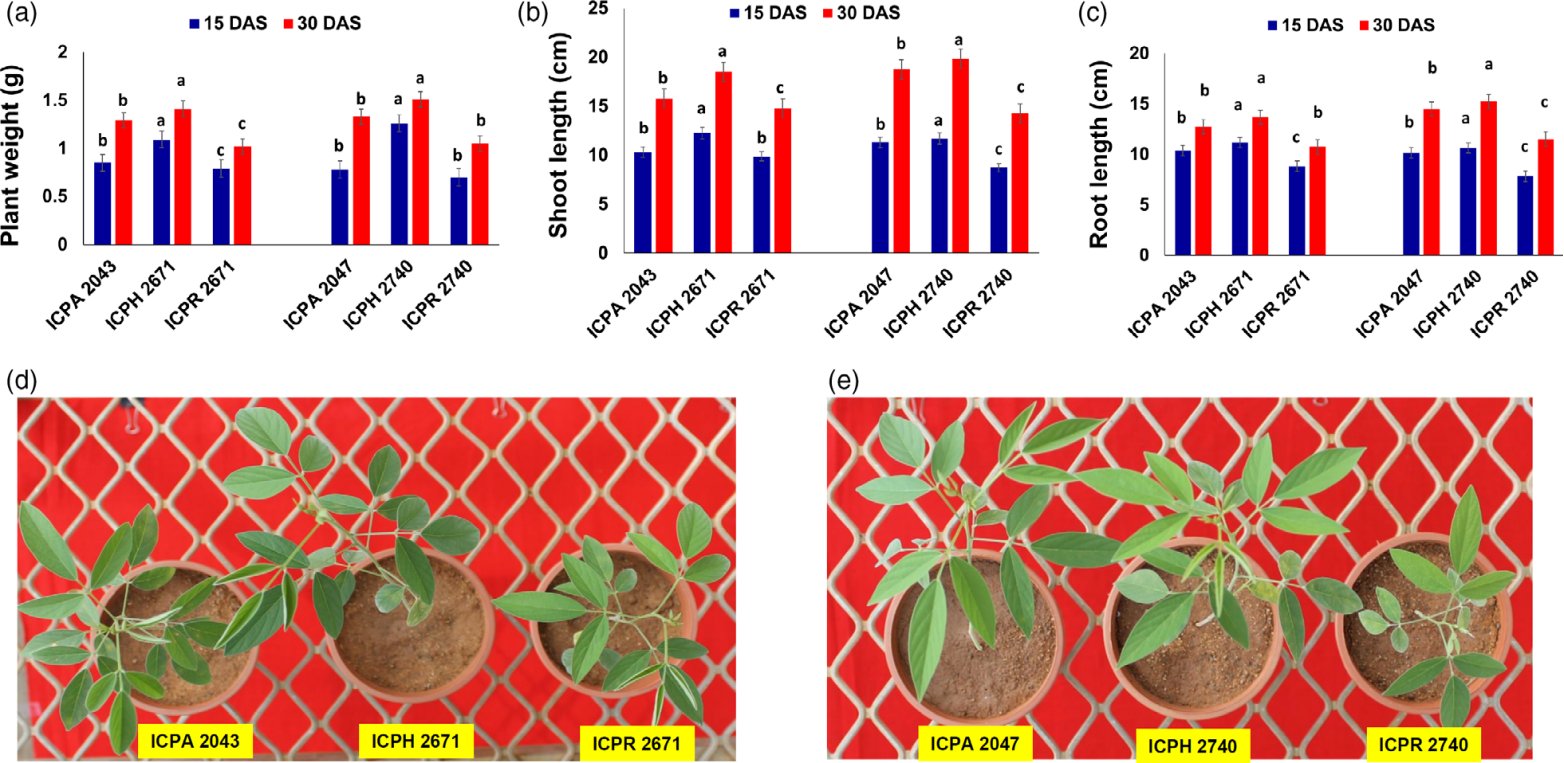
Hybrids showed differences in their vegetative growth patterns and levels of heterosis. (a–c) Levels of vegetative heterosis represented as the difference in (a) plant weight, (b) shoot length and (c) root length between the hybrid and parental lines at 15 and 30 DAS time points. Duncan's analysis was employed to test statistical significance among the classes. Different alphabets indicated in the graphs revealed significant differences between the groups at *P* < 0.05 level of significance. All error bars represent SEM. (d) Seedlings of ICPH 2671 and parental lines showing increased vegetative growth in F_1_ hybrid compared with the parental lines (e) seedlings of ICPH 2740 and parental lines showing increased vegetative growth in F_1_ hybrid compared with the parental lines.

Two commercially released hybrids namely, ICPH 2671 (crossed between ICPA 2043; CMS line and ICPR 2671; restorer line) and ICPH 2740 (crossed between ICPA 2047; CMS line and ICPR 2740; restorer line), and their parental lines were utilized in the present study to understand the possible mechanism underlying heterosis. Strong heterosis was observed at the early seedling stages of vegetative growth in pigeonpea. We compared the biomass heterosis in terms of plant weight, root length and shoot length in the hybrids, ICPH 2671 and ICPH 2740 with their parental lines, ICPA 2043/ICPR 2671 and ICPA 2047/ICPR 2740, respectively at both 15 and 30 days after sowing (DAS) (Figure 2a,c). The mid‐parent value (MPV) was calculated from the plant weight, root length and shoot length (Table S1). To establish the timing of heterosis onset in pigeonpea, we phenotyped three growth parameters namely, plant weight, shoot length and root length at two different growth stages, *viz.,* 15 and 30 days after sowing (DAS) (Figure 2d,e, Table S1, Appendix S1). We compared MPV to that of hybrids. At 15 DAS higher plant weight (19.51% and 21.62% increase over MPV for ICPH 2671 and ICPH 2740, respectively), higher shoot height (21.56% and 16.85% increase over MPV for ICPH 2671 and ICPH 2740, respectively) and greater root length (16.53% and 18.50% increase over MPV for ICPH 2671 and ICPH 2740, respectively) were observed in hybrids. The same increase was observed in all three growth parameters at 30 DAS. We found that heterosis is established relatively early in the seedling stage and hence, we used 15 DAS plants in further analysis.

### Global methylation profile of hybrids and their parental lines

**Figure 3:**
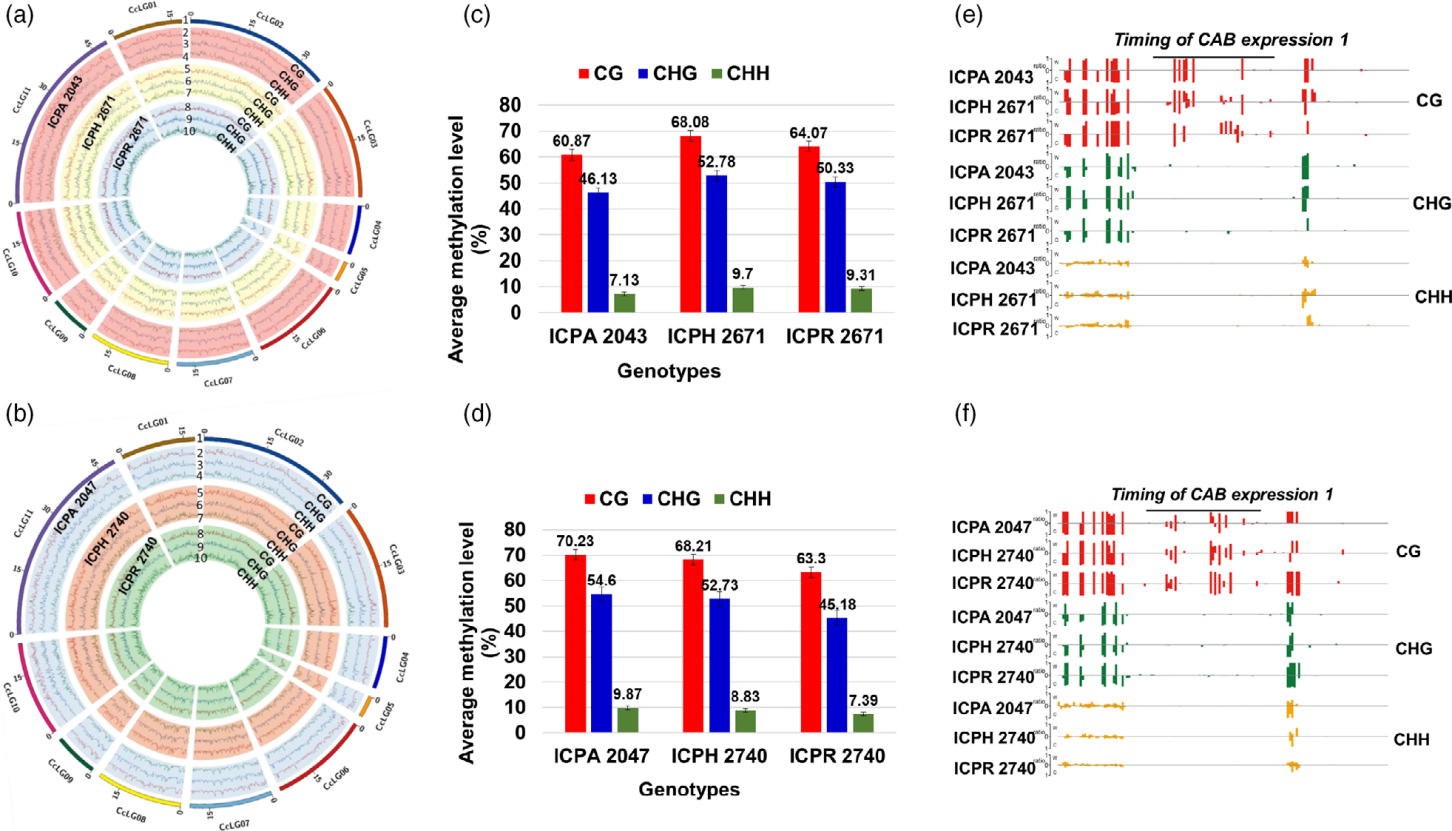
Global methylome maps and DNA methylation landscapes of hybrids and parental lines. (a–b) There are ten circles (label 1–10), the outermost circle (1) represents 11 pseudomolecules of *Cajanus cajan* and three types of methylation in CMS line (2–4), F_1_ hybrid (5–7) and restorer line (8–10). The order of features from inside to outside is (1) CHH, (2) CHG, (3) CG for (a) ICPH 2671 and (b) ICPH 2740. Elevated DNA methylation in (c) ICPH 2671 and (d) ICPH 2740 relative to their parental lines. Columns represent bulk methylation levels at three cytosine contexts in the hybrid and parental lines as determined by bisulfite sequencing. An example of DNA methylation profiles at three cytosine contexts in a representative region of the gene *Timing of CAB expression 1* (*TOC1*) in (e) ICPH 2671 and (f) ICPH 2740 and their parental lines. The height of the bar is proportional to the number of reads detected on each strand.

To explore the role of epigenetic regulation in heterosis, we generated single‐base resolution maps of methylated cytosines by using bisulfite sequencing of 15 DAS seedlings for six genotypes which includes two hybrids and their parental lines (Appendix S1, Figure 3a,b, Table S2, Figures S1 and S2). In general, there was a significant (*P*‐value <0.001) increase in DNA methylation in hybrids (8.91% in ICPH 2671 and 9.60% in ICPH 2740) when compared to the MPV (Figure 3c–f, Appendix S1, Table S3, Figures S3–S7). Comparative analysis of per cent methylation contributed from parental lines in hybrid revealed 85.56% (ICPH 2671) and 88.87% (ICPH 2740) loci in hybrids where methylation was contributed by both the parents. It was found that 3.86% and 5.63% of methylation was uniquely contributed from ICPA 2043 and ICPR 2671 respectively in the hybrid ICPH 2671 (Figure S8a). Similarly, ICPA 2047 and ICPR 2740 contributed methylation of 2.01% and 2.23% uniquely to the hybrid ICPH 2740 (Figure S8b). Interestingly, it was noted that at 2.69% (ICPH 2671) and 1.50% (ICPH 2740) loci, there was no methylation present in hybrids, but either of the parents was methylated.

### Thousands of DMRs are detected between hybrid and their parental lines

Many DMRs were identified between parental lines and hybrids of both the hybrid combinations (Appendix S1, Figure S9). A significant number of non‐redundant sets of DMRs between the parents and hybrids (13 987 for ICPH 2671 and its parental lines and 15 132 for ICPH 2740 and its parental lines) were observed (Table S4). Among the identified DMRs, methylation levels in 96.59% (13 511; ICPH 2671) and 97.38% (14 736; ICPH 2740) of DMRs were significantly different (either higher or lower) from MPVs, suggesting methylation interaction in a non‐additive manner (interactive (I) DMRs) at these regions (Appendix S1, Table S4). Whereas less percentage (3.41% in ICPH 2671 and 2.62% in ICPH 2740) of DMRs were classified as non‐interactive (NI) which were present in additive manner. We observed an increased number of DMRs enriched 2 kb upstream and 2 kb downstream of protein‐coding genes, whereas there were fewer DMRs located within gene (Appendix S1, Table S5). DMRs present within gene or 2 kb flanking regions were considered as gene associated DMR. Overall, 26.99% and 24.58% were found to be gene associated DMR in ICPH 2671 and ICPH 2740, respectively (Table S5).

Differentially methylated regions were further classified as transchromosomal methylation (TCM), in which the methylation level in hybrids was significantly higher than MPVs (FDR < 0.01), and transchromosomal demethylation (TCdM), where the methylation level of hybrids was considerably lower than MPVs (FDR < 0.01). Of the 13 512 DMRs, there were 7844 TCM DMRs and 5668 TCdM DMRs for ICPH 2671 with a significant difference with MPVs (Table S6). In the case of ICPH 2740 out of 14 737 DMRs, 6269 and 8468 were classified as TCM and TCdM DMRs, respectively, and it was noticed that both high‐parent and low‐parent alleles have contributed to the increased and decreased methylation levels in hybrids (Table S6). It was noted that in all the three cases [NI and I (TCM and TCdM)] DMRs were enriched within intergenic (2 kb upstream, 2 kb downstream) followed by genic and the transposable element of gene regions.

### Association of 24‐nt siRNA clusters with DNA methylation

We have generated ~143 million reads (49 bp per read) from the six  sRNA‐seq libraries, and approximately 5 million unique sRNAs were identified for all the six genotypes (Appendix S1, Tables S7 and S8, Figures S10 and S11). Class distribution analysis of filtered reads showed that 21‐ and 24‐nt classes were the most abundant groups in both the hybrid combinations (Figure S10a,b). In both the hybrid combinations, a large number of 24‐nt siRNA clusters mapped to the intergenic regions (ranging from 71.1% to 78.9%). The non‐TE‐related genes (ranging from 17% to 18.6%) showed more enrichment of 24‐nt siRNA cluster than in TE‐related genes (ranging from 4.2% to 6.3%) (Figure S11). The integration of genomic coordinates of 24‐nt siRNA clusters with the pigeonpea genome annotation revealed ~12% of 24‐nt siRNA clusters were originated from the genic and flanking sequences for both the hybrids (Table S8).

#### 24‐nt siRNA clusters were associated with increased DNA methylation in hybrids

**Figure 4:**
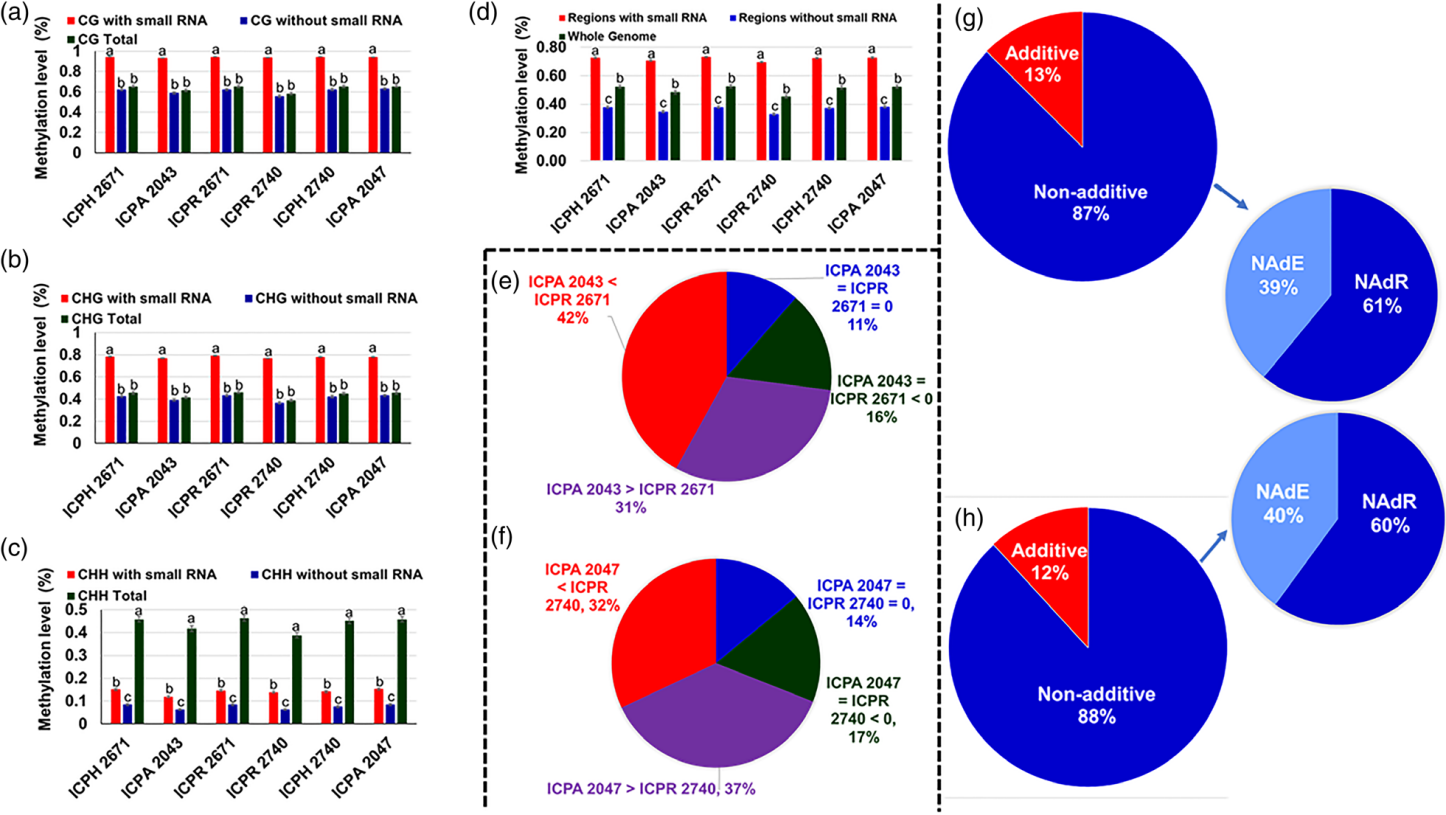
The genomic distribution of 24‐nt siRNAs clusters in hybrids and its expression. Regions covered by 24‐nt siRNA account for most of the methylation increase in CG (a), CHG (b), and CHH (c) cytosine contexts in hybrids. (d) Contribution to increased DNA methylation in F_1_ hybrids by the regions with and without 24‐nt siRNA in comparison with the whole genome. Duncan's analysis was employed to test statistical significance. Different alphabets indicated in the graphs revealed significant differences between the groups at *P* < 0.05 level of significance. (e–f) Contribution to increased DNA methylation in F_1_ hybrids by the regions with or without  24‐nt siRNA. The cytosine positions of the genome were divided into four categories based on the methylation levels of the parental lines (i) positions highly methylated in CMS line than restorer line (ICPA 2043 > ICPR 2671; ICPA 2047 > ICPR 2740), (ii) positions highly methylated in restorer line than CMS line (ICPR 2671 < ICPA 2043; ICPR 2740 < ICPA 2047), (iii) positions where methylation was detected but levels were equal in CMS and restorer lines (ICPA 2043 = ICPR 2671 > 0; ICPA 2047 = ICPR 2740 > 0) and (iv) positions lacking detectable methylation in both CMS and restorer lines (ICPA 2043 = ICPR 2671 = 0; ICPA 2047 = ICPR 2740 = 0). (g–h) Non‐additive expression of 24‐nt siRNA clusters in hybrids. Comparative analysis of 24‐nt siRNA clusters differentially expressed between ICPH 2671 (g) and ICPH 2740 (h) combinations, respectively.

We analysed our data to investigate the relationship between 24‐nt siRNA and DNA methylation. It was found that DNA methylation levels were significantly higher in the regions with sRNA than those without sRNA in both the hybrid combinations (Figure 4a–h). To investigate a possible role of 24‐nt siRNAs in hybrid methylome interactions, we investigated the presence of 24‐nt siRNA clusters in TCM and TCdM DMRs (as mentioned earlier). In the case of ICPH 2671, 24‐nt siRNA clusters were found in 80.72% of TCM DMRs and 83.51% of TCdM DMRs (Table S9). Similarly, in the case of ICPH 2740, we observed that 24‐nt siRNA clusters were present at 77.71% of TCM DMRs and 78.22% of TCdM DMRs (Table S10), respectively. The presence of 24‐nt siRNA clusters followed a pattern of higher siRNA in CHH methylation followed by CG and CHG methylation in both the hybrids. Our analysis revealed that in ICPH 2671, 86.44% (TCM) and 85.18% (TCdM) of 24‐nt siRNA clusters were contributed equally from both the parents. It was noted that a smaller number of 24‐nt siRNA clusters (~4% to 5%) were present uniquely in parents and ~37%–50% of 24‐nt siRNA clusters were contributed from the parents in ICPH 2671 (Table S9). In ICPH 2740, 83.53% (TCM) and 85.64% (TCdM) of 24‐nt siRNA clusters were contributed equally from both the parents. There were ~3%–7% 24‐nt siRNA clusters that were uniquely present in the parental lines, and ~40%–60% of 24‐nt siRNA clusters were contributed from the parents in the hybrid ICPH 2740 (Table S10). Additionally, there were few DMR positions (~1%–3%) in both the hybrids where 24‐nt siRNA clusters were present only in hybrid and not in the parental lines. This uniquely present 24‐nt siRNA clusters in hybrids could be due to their trans‐generation from one parent leading to trigger DNA methylation in hybrids. Further, classification of small 24‐nt siRNA clusters according to the methylation level in parental lines revealed regions covered by 24‐nt siRNA clusters that were differentially methylated in the parents contributed 72.9% and 69.0% of the increased methylation in ICPH 2671 and ICPH 2740, respectively (Appendix S1, Table S11, Figure 4e,f). This indicates the mobility of 24‐nt siRNA clusters that mediate epigenetic regulation renders them promising candidates for modulating transgressive phenotypes in hybrids. These results suggested that the 24‐nt siRNA might be associated with DNA methylation interactions in DMRs.

#### Non‐additive expression of 24‐nt siRNA clusters in hybrids

To characterize the effects of 24‐nt siRNA clusters on gene expression, we surveyed differential expression of the 24‐nt siRNA clusters between hybrids and parental lines. Comparative analysis of hybrids and parental lines identified 87.6% and 88.1% of the 24‐nt siRNA clusters differentially expressed between ICPH 2671 and ICPH 2740 combinations, respectively (Figure 4g,h). Results showed that a higher number of non‐additively expressed 24‐nt siRNA clusters were present in both the hybrids. It was noted that in ICPH 2671, the number of 24‐nt siRNA clusters with an expression level higher than the MPV (39%) was significantly higher than the number of 24‐nt siRNA clusters with an expression level lower than the MPV (61%) (*P* ≤ 0.001). However, in the case of ICPH 2740, the number of 24‐nt siRNA clusters with an expression level higher than the MPV (40%) was significantly lower than the number of 24‐nt siRNA clusters with an expression level lower than the MPV (60%).

### Expression dynamics of miRNA in hybrids

To understand the expression dynamics of miRNA, miRNA‐enriched genomic regions were identified in the two hybrids and their parental lines. In addition to the known miRNAs deposited in the miRbase database, we identified 1289 novel miRNAs having 15 316 targets in the selected six genotypes. A total of 702 conserved miRNAs belonging to 145 families identified in the selected six genotypes. Out of the 702 conserved miRNAs, 545 and 601 were found to be non‐additively expressed (*P* ≤ 0.05, FDR ≤ 0.05) between ICPH 2671—parental lines and ICPH 2740—parental lines, respectively. Of the 545, 274 were non‐additively repressed and 252 were non‐additively activated in ICPH 2671 combination. In ICPH 2740 combination, 353 and 248 were non‐additively repressed and non‐additively activated, respectively. The target genes of these non‐additively expressed miRNAs were predicted from the gene models in the pigeonpea genome annotation (Varshney *et al.*, 2012). A total of 591 target transcripts were predicted for 305 of the 702 conserved miRNAs. Based on the identified significance of the expressed miRNA 61 and 92 differentially expressed miRNA (DESs) between hybrid and MPV were identified in ICPH 2671 and ICPH 2740 hybrid combinations, respectively (Table S12). We further investigated the correlation between differentially expressed miRNAs and the expression of their targets. There was significant negative correlation between the expression level of 29 (47.5 %; *r* = −0.66, *P*‐value <0.01) and 41 (44.5 %; *r* = −0.72, *P*‐value <0.01) differentially expressed miRNA and corresponding target genes.

### More genes were actively expressed in the hybrids compared to their parents

*De‐novo* assemblies of hybrids and their parental lines were obtained from 260.28 million paired‐end reads of mRNA sequencing data (Table S13). As a result, a total of 53 996 unigenes were annotated. Further the unigenes were utilized for identification of differentially expressed genes (DEGs) (Appendix S1, Tables S14–S16, Figures S12–S14). To identify DEGs, the developed *de novo* assemblies were compared in all possible combinations (CMS/female parent vs F_1_/hybrid, Restorer/male parent vs F_1_/hybrid, CMS/female parent vs Restorer/male parent) (Table S17, Figures S15–S17) and between the hybrids as well (Figure S18). Additionally, to identify the potential DEGs associated with heterotic phenotype, we compared hybrids with MPV designated as hybrid‐MPV DEGs. The genes following *P* ≤ 0.05, FDR with ≤0.001 and log 2 ratios with ≥1 were scored as DEGs in all the pairwise comparisons (Table S17). A significant number of DEGs were observed between each hybrid and their corresponding parental lines. Many DEGs were also identified between the hybrids (ICPH 2671 and ICPH 2740) suggesting the presence of a significant difference between the two hybrid combinations. Further, global transcriptome analysis of the two hybrids and their parents revealed all possible modes (additive and non‐additive) of gene actions in hybrids (Appendix S1, Table S18, Figure S19a,b).

**Figure 5:**
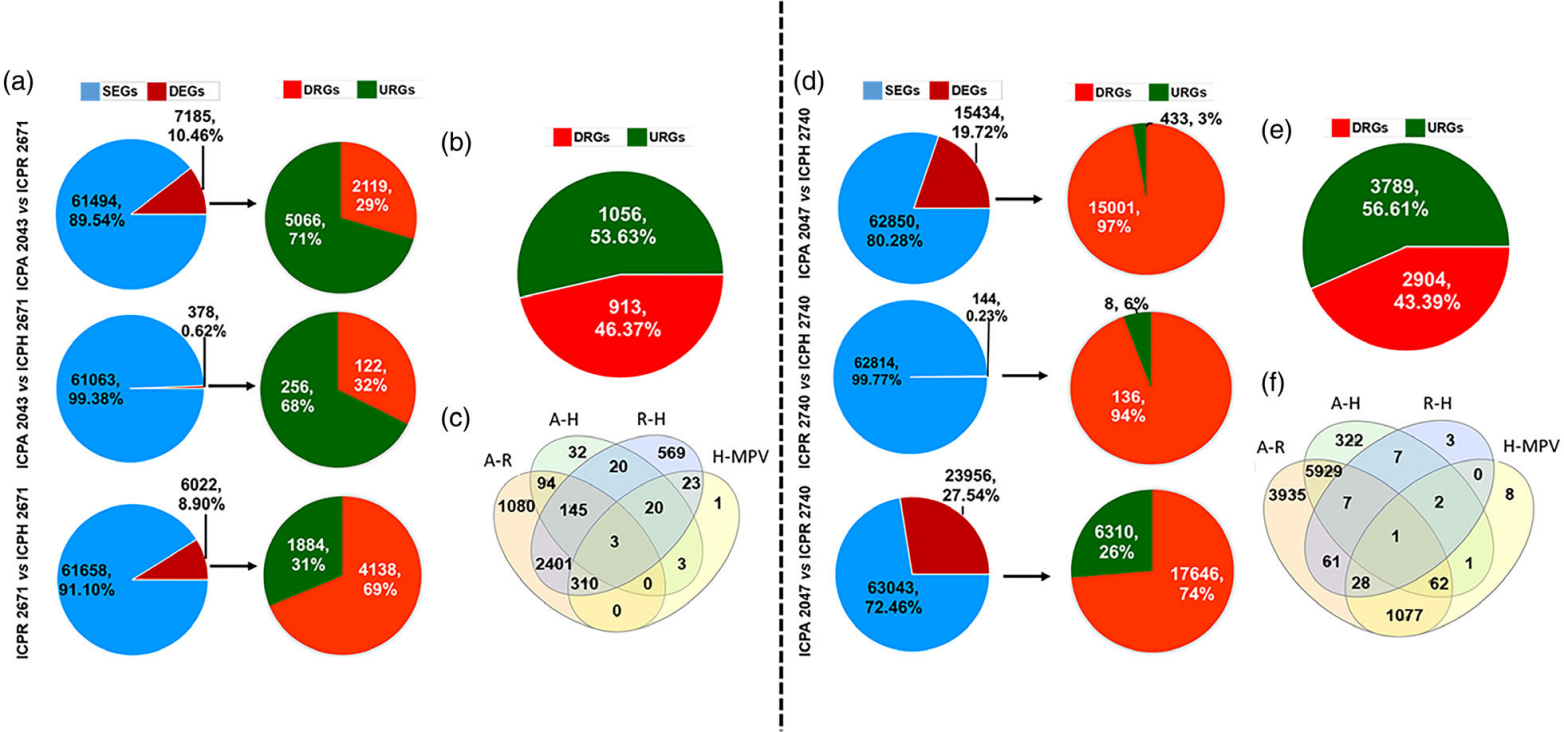
Transcriptomes of hybrid and parental lines. (a) The number of differentially expressed (DEGs) and similarly expressed genes (SEGs) and further DEGs were further classified as down‐regulated genes (DRGs) or up‐regulated genes (URGs) based on the log_2_fold expression values between ICPA 2043 vs ICPR 2671 (parental line combinations), ICPA 2043 vs ICPH 2671 (CMS lines vs hybrid), ICPR 2671 vs ICPH 2671 (restorer line vs hybrid). (b) classification of hybrid‐MPVsDEGs in DRGs and URGs. (c) Venn diagram showing all of the differentially expressed genes (DEGs) in ICPH 2671 combinations namely, A‐R (ICPA 2043 vs ICPR 2671), A‐H (ICPA 2043 vs ICPH 2671), R‐H (ICPR 2671 vs ICPH 2671) and H‐MPV (hybrid‐MPVs DEGs). (d) The number of DEGs and SEGs classified in ICPH 2740 combinations. Identified DEGs were classified as DRGs or URGs based on the log_2_fold expression values between ICPA 2047 vs ICPR 2740 (parental line combinations), ICPA 2047 vs ICPH 2740 (CMS lines vs hybrid), ICPR 2740 vs ICPH 2740 (restorer line vs hybrid). (e) classification of hybrid‐MPVsDEGs in DRGs and URGs. (f) Venn diagram showing all of the differentially expressed genes (DEGs) in ICPH 2740 combinations namely, A‐R (ICPA 2047 vs ICPR 2740), A‐H (ICPA 2047 vs ICPH 2740), R‐H (ICPR 2740 vs ICPH 2740) and H‐MPV (hybrid‐MPVsDEGs).

We compared the transcriptome of parents of hybrid ICPH 2671, and a total of 7185 (10.46% of total expressed genes) DEGs were observed between ICPA 2043 and ICPR 2671 (Figure 5a). In this combination, most of the genes were found to be up‐regulated (70.51%). Further, we identified DEGs between hybrid and parental lines. As a result, 378 (0.62%) and 6022 (8.90%) DEGs were observed between ICPA 2043/ ICPH 2671 and ICPR 2671/ICPH 2671, respectively. It was observed that a higher percentage of DEGs (67.72%) were up‐regulated between ICPA 2043 and ICPH 2671, whereas between ICPR 2671 and ICPH 2671 more percentage of DEGs (68.71%) were down‐regulated (Figure 5a). Further, we looked for hybrid‐MPV DEGs and it was noted that only a small fraction (1969, ~3%) of genes were expressed non‐ additively in hybrid. Out of the identified 1969 hybrid‐MPV DEGs, more number of genes were down‐regulated (53.63%) as compared to up‐regulated (46.37 %) genes in hybrid (Figure 5b). It was noted that ~25% of DEGs were common between the parental lines (ICPA 2013–ICPR 2671) and hybrid‐MPV. Interestingly, among the selected four combinations of DEGs, none of the DEGs were common among the four combination of DEGs (Figure 5c).

In ICPH 2740 hybrid combination, comparison of expressed transcripts between parental lines (ICPA 2047/ICPR 2740) identified 23, 956 (27.54% of total expressed genes) DEGs (Figure 5d). similar to ICPH 2671 hybrid results, ICPH 2740 also has higher number of up‐regulated (73.66%) DEGs. Further, identification of DEGs between hybrid and parental lines revealed, 15434 (19.72%) and 144 (0.23%) DEGs between ICPA 2047/ICPH 2740 and ICPR 2740/ICPH 2740 respectively. It was observed that a higher percentage of DEGs (97.19% and 94.44%) were up‐regulated between ICPA 2047/ICPH 2740 and ICPR 2740/ICPH 2740. A total of 6693 (~11%) DEGs were identified as hybrid‐MPV in ICPH 2740 hybrid combination, which was significantly higher than ICPH 2671 hybrid‐MPV DEGs (~3%). Out of 6693 hybrid‐MPV DEGs, higher number of genes were up‐regulated (56.61%) as compared to down‐regulated genes in hybrid (43.39%) (Figure 5e). It was found that ~16% of DEGs were common between the parental lines (ICPA 2047–ICPR 2740) and hybrid‐MPV. Comparative analysis of all four set of DEGs revealed 1077 DEGs were common among them (Figure 5f).

### Divergent DNA methylation patterns influenced gene expression

To understand the influence of DNA methylation on the expression of genes, we retrieved information of genes which are differentially expressed and are methylated (designated as differential methylated genes, DMGs). We identified two types of DMGs (i) *hybrid and parental lines DMGs* and (ii) *hybrid‐MPV DMGs* in genic and flanking regions (2 Kb upstream and 2 Kb downstream) of the genes. Analysis of hybrid and parental lines identified a total of 1162, 46 and 765 DMGs (genic and flanking regions) identified between ICPA 2043/ICPR 2671, ICPA 2043/ICPH 2671 and ICPR 2671/ICPH 2671, respectively. Similarly, 3521, 1826 and 12 DMGs were observed between ICPA 2047/ICPR 2740, ICPA 2047/ICPH 2740 and ICPR 2740/ICPH 2740, respectively (Table S19). It was noted that a higher level of DMGs was identified in the flanking regions (2 Kb up and 2 Kb down) of genes compared to within the genic region. The average expression of genes was lower in the genic as compared to flanking DMG. Hybrid‐MPV DMGs analysis identified that among 1969 hybrid‐MPVs DEGs identified in ICPH 2671 combination, 131 (~6.65%) were DMGs whereas, among 6691 hybrid‐MPVs DEGs identified in ICPH 2740 combination, 599 (~8.95%) were DMGs.

### Insight into heterosis in pigeonpea hybrids

We generated multi‐omics data and performed phenotypic analysis in two commercially released hybrids to understand the molecular mechanism involved in pigeonpea heterosis (Figure S20).

#### Role of hybrid‐MPV DEGs and DMGs in heterosis

We presume genes in a hybrid that had expression level significantly different from the MPV (*P*‐value <0.05) could potentially be responsible for generating the heterotic phenotypes in hybrids. Gene enrichment analysis of 131 and 599 DMGs (methylated‐hybrid‐MPVs DEGs) identified 73 and 224 significantly enriched GO functions terms (*P*‐value <0.05) in ICPH 2671 and ICPH 2740, respectively. We used ReViGO (reduced and visualized gene ontology) to cluster the significantly overrepresented GO terms (Table S20).

**Figure 6:**
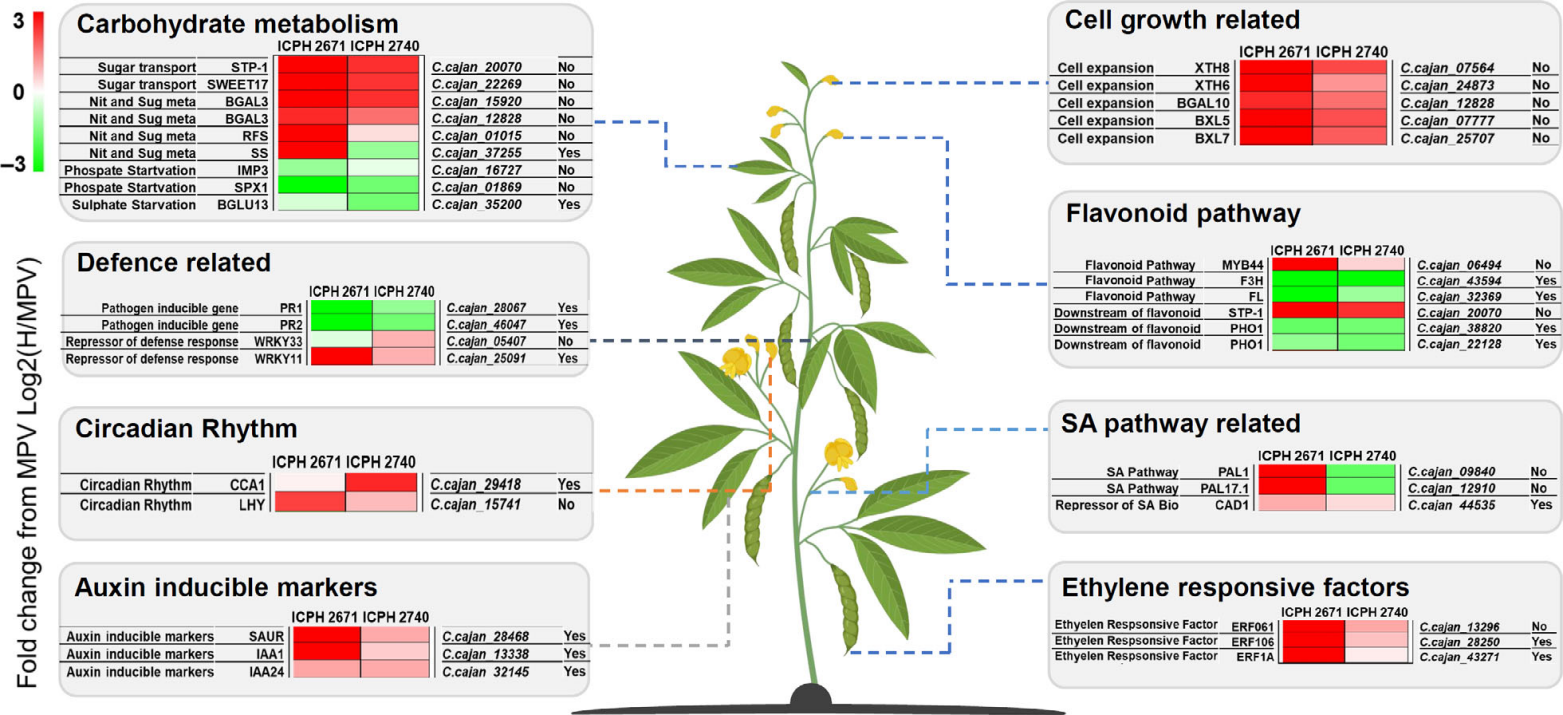
Role of differentially expressed genes (DEGs) and differentially methylated genes (DMGs: methylated‐hybrid‐MPV DEGs) in hybrids. DEG patterns in several key regulatory networks associated with heterosis including, metabolism, plant growth, circadian rhythm, defence and stress response, are presented for both the hybrids. DMGs (methylated‐DEGs) are denoted in logical as Yes/No. The repressed expression of methylated genes resulting in altered response in defence, SA pathway and flavonoid pathway. The logarithmic transformation of FPKM of hybrids with MPV denotes the level of expression in the figure. Genes associated with sugar transport and nitrogen and sugar metabolism (*STP‐1, SWEET17, BGAL3*) were up‐regulated in both the hybrids while *RFS* and *SS* were only up‐regulated in ICPH 2671. Phosphate and sulphate starvation‐induced genes (*IMP3, SPX1, BFLU13*) showed a down‐regulated expression pattern in both the hybrids. Inverse gene regulation between expressor (*PR1, PR2*) and repressor (*WRKY33, WRKY11*) of defence response genes was observed in hybrids. *CC1* and *LHY* showing positive regulation in clock cycle, ethylene responsive factors, auxin‐responsive genes, and growth regulated genes are indicating an increased expression in both the hybrids alternate expression of genes relating to flavonoid and SA pathway showing the response in two hybrids.

DEGs associated with abiotic stimuli and stresses identified 15 and 7 significantly enriched GO terms for ICPH 2671 and ICPH 2740 hybrid combinations, respectively (Table S20). Enrichment of the terms associated with the above‐mentioned process reflected greater energy production and a broader tolerance to environmental conditions in hybrids as compared with the parental lines (Figure 6). Genes associated with sugar transporters (*STP1* and *SWEET17*) and nitrogen and sugar metabolism (*SS, RS* and *BGAL3)* were found to be mostly up‐regulated in both the hybrids. The down‐regulation of phosphate starvation‐induced genes (*IMP3* and *SPX1*) and sulphate starvation‐induced gene (*BGLU13*) in hybrids compared with MPV is a transcriptional state that could be related to greater plant growth. Among the up‐regulated genes in hybrids as compared to MPV, included key regulators of circadian clock (*CCA1* and *LHY*).

Biological processes associated with biotic defence response were the most prevalent GO terms associated with DEGs in both the hybrids. A total of 9 and 35 significantly enriched GO terms identified associated with biotic defence response for ICPH 2671 and ICPH 2740 hybrid combinations, respectively. Among the down‐regulated defence responsive genes, there are several genes that are well known to be induced by pathogen attack including *PR1* and *PR2. WRKY TRANSCRIPTION FACTOR (WRKY11)* associated with defence response, known to function as repressor of plant defence were found up‐regulated in both the hybrids. However, *WRKY33* was up‐regulated in ICPH 2740 and down‐regulated in ICPH 2671. Changes in biotic defence response were the principal themes associated with the transcriptional changes occurring in both the hybrids. The pattern of altered defence‐related gene expression implies that the hybrids have a decreased basal defence response, this being more pronounced in ICPH 2740 as compared to ICPH 2671.

Hormones are important regulators of plant growth and response to defence. GO enrichment analysis showed altered gene expression changes in both the hybrids as compared to MPV. Auxin (indole acetic acid; IAA) and Salicylic acid (SA) are the two essential hormones known to control plant growth as well as stress and defence response. IAA is derived mainly from tryptophan through several key intermediates. However, in both of the hybrids in our study, we could not find any altered expression in genes related to auxin pathway as compared to their MPVs. Whereas, an up‐regulation in genes related to auxin‐inducible marker (*IAA1, IAA24* and *SAUR*) was observed in both the hybrids which reflect the state of the increased auxin level in hybrids. Additionally, genes related to flavonoid pathways (repressor of auxin transport) namely, *F3H* and *FL* were found down‐regulated in both the hybrids except, *MYB44* which were found to be up‐regulated in both the hybrids. Gene associated with downstream target of flavonoids has been found up‐regulated (*STP1*), whereas genes associated with downstream target of IAA have been found to be down‐regulated (*PHO1*) in ICPH 2671 but not found differentially regulated in ICPH 2740.

Salicylic acid (SA) is another important hormone associated with plant defence responses, abiotic stresses tolerance and plant growth. SA is derived mainly from two pathways (IC isochorismate pathway and PHE; phenylalanine pathway). The IC pathway is predominant pathway for SA biosynthesis compared with PHE pathway. We have not found any genes associated with IC pathway (*ICS1* and *ICS2*), which were differentially regulated in both of the hybrids compared with MPVs. However, two genes associated with PHE pathways (*PAL1* and *PAL17.1*) were found differentially up‐regulated in ICPH 2671 and down‐regulated in ICPH 2740 compared with MPV. Gene associated with repressor of SA biosynthesis was found to be up‐regulated (*CAD1*). SA regulated genes controlling cell expansion were found up‐regulated (*XTH 6*, *XTH8, BXL 5, BXL7* and *GAL3*) in both the hybrids. Similarly, SA repressed genes that were up‐regulated in both the hybrids are several *ETHYLENE RESPONSE FACTORS* (ERF). Overexpression of *ERF1* induced salt, drought and heat stress tolerance in plants. *ERF061* and *ERF106* associated with plant‐specific transcription factor that activates the expression of abiotic stress‐responsive genes.

#### Role of DESs in heterosis

**Figure 7:**
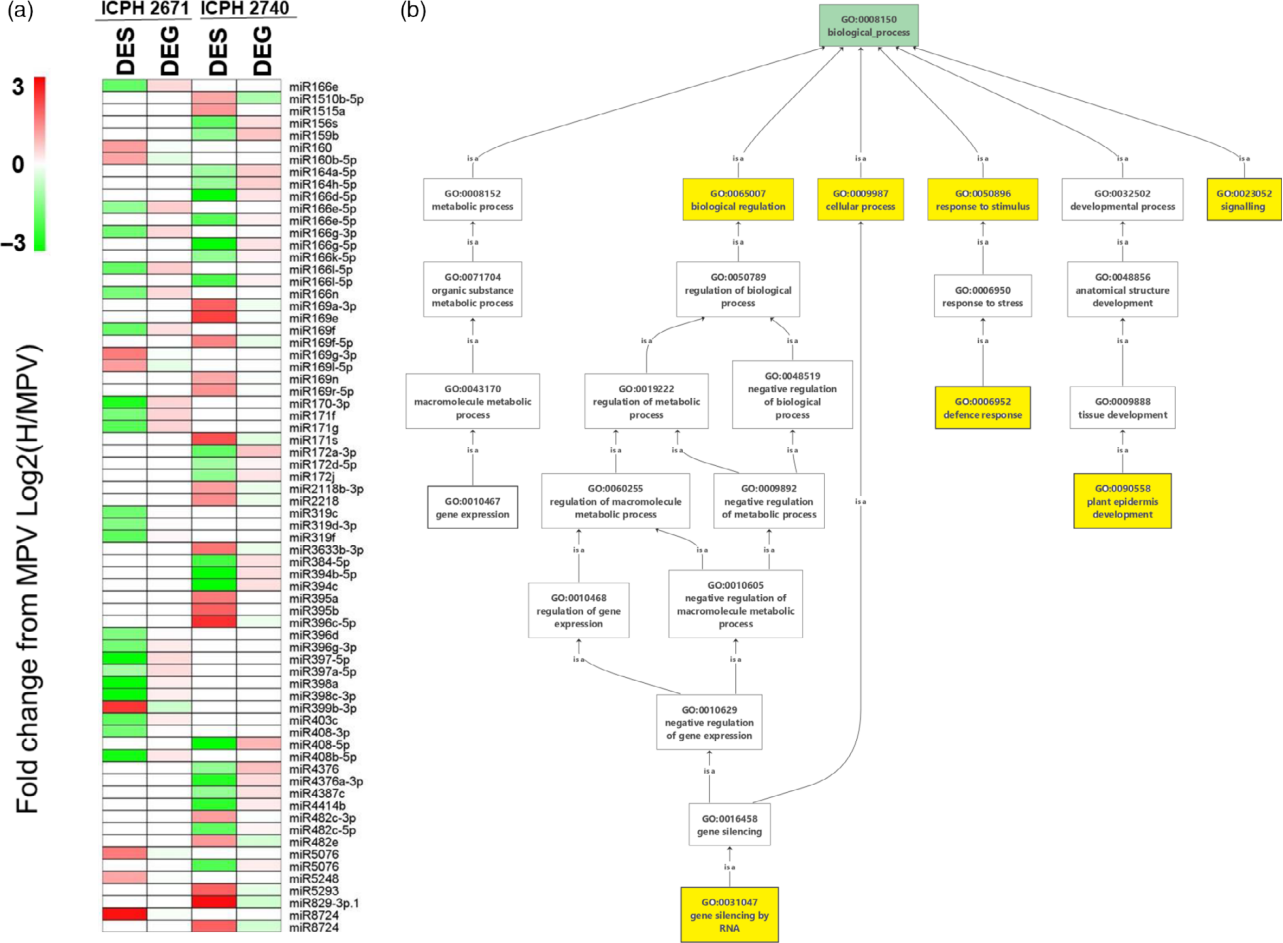
Contribution of miRNA in heterosis. (a) Comparison of differentially expressed small RNAs (DESs) and their corresponding differentially expressed genes (DEGs) at their expression level. miRNA of both common and unique to hybrids are projected. There are 32 miRNA families identified as DES in ICPH2671 and ICPH2740. Sixteen miRNA families of 29 miRNA in ICPH2671 and 26 families of 41 miRNA are in ICPH2740. Seven miRNA families found in both the hybrids (miR166, miR169, miR171, miR396, miR408, miR8724 and miR5076). Common miRNAs represent themselves as families in two hybrids, but still have unique targets while targeting at the structural level. log transform of FPKM values is being denoted in colours from green to red (low to high). (b) GO enrichment analysis of miRNA targets and yellow colour represents the GO term enriched with significant value of *P* ≤ 0.01, showing miRNA playing roles in functions like RNA silencing, growth and signalling, defence response, plant cell, development and cellular process.

A total of 29 and 41 DES are obtained in ICPH2671 and ICPH2740, respectively. The pattern of DES's and DEGs clearly indicates the support of heterosis by activating the genes responsible for plant growth, cell growth and differentiation, stress response, seed germination by its own down‐regulation and suppressing the gene expression involving in ion binding, defence response and negative regulators of auxin signalling pathway. We have observed that seven commonly obtained miRNA families (miR166, miR169, miR171, miR396, miR408, miR8724, miR5076) do not share the same targets, specifically miRNA from the same family expresses only in any one of the hybrids (Table S12, Figure 7). They have potentially different targets in all the five common miRNA families. Even though miRNA is conserved in nature, and it is not necessary for miRNA to function in the same manner. We observed that the targets of miRNA are different but the molecular function of targeted genes shows some similarity inside the miRNA families. For example in miR166 family, miR166e‐5p, miR166g‐3p, miR166g‐3p, miR166l‐5p and miR166n targets *PRE3*, and *ATHB‐15* while another miRNA from the same family targets *PRE3* and *MLO3* genes; hence, all the three genes targeted by the miR166 family are DNA binding activity and though, the biological process are different among the three genes they all contribute to the cell growth and differentiation. Thus, the expression of miR166 family is down‐regulated in order to act as a positive regulator of heterosis. miR169 and miR399 are up‐regulated as their targeted genes are involved in ion binding activity correspond to induce stress. Differential targeted genes of miRNA maintain the regulation of heterosis with similar biological process and molecular functions. The important genes mainly contributing to plant growth and development, defence response, stress responses, seed germination are altered by RNA interference. Negative co‐regulation of gene expression between miRNA and mRNA interferes with the physiological interaction pathways resulting in increased morphological change.

## Discussion

### Increased DNA methylation changes in hybrids uncover the role of 24‐nt siRNA clusters

Our analysis reveals large‐scale DNA methylation changes in hybrids as compared to their parental lines, in general increased DNA methylation in hybrids. A large number of DMRs are detected in both the hybrids. DMRs are more enriched in intergenic region than in genic region, indicating their likely regulatory role in heterosis (He *et al.*, 2010). DNA methylation was found predominantly in transposable elements and other repetitive regions. A stable repressive epigenetic mechanism in these regions maintains genome stability by suppressing their activity (Chan *et al.*, 2005; Law and Jacobsen, 2010). We also found that DNA methylation was present in the promoter and transcribed regions of protein‐coding genes, suggesting their potential role in regulating gene activity (Greaves *et al.*, 2012; Jin *et al.*, 2008; Zhao *et al.*, 2007).

In the present study, the methylated loci were frequently accompanied by 24‐nt siRNA clusters in both the hybrids. Approximately one‐third of the methylated loci was rich in 24‐nt siRNA clusters, supporting their essential role in DNA methylation (Shen *et al.*, 2012). Differences in the presence of 24‐nt siRNA clusters in the parents at specific loci could lead to the changes in hybrids, which, in turn, could lead to hyper‐ (TCM‐DMRs) and hypo‐methylations (TCdM‐DMRs) (Groszmann *et al.*, 2011). Our results showed an association of TCM‐DMRs and TCdM‐DMRs with 24‐nt siRNA clusters. Genomic regions that undergo TCdM usually were associated with decreased 24‐nt siRNA expression in comparison with TCM DMRs. In our study, the TCdM DMRs involve decreases in CHH methylation, followed by CHG and CG methylation in both the hybrid combinations. The differences in loss of methylation between the cytosine methylation contexts are presumed to be a result of differences in the pathways maintaining each context. Therefore, our data indicated that 24‐nt siRNA clusters are critical for increased DNA methylation in hybrids.

### Differential gene regulation and methylation in hybrids play an essential role in heterosis

High‐quality transcriptome assembly is the crucial first step for analysing the molecular basis of phenotypes of interest (Martin and Wang, 2011). Therefore, we have developed a high‐quality *de‐novo* assembly of the two selected hybrids and their respective parents and identified several novel transcripts in our developed *de‐novo* assembly which was not reported before in the available pigeonpea transcriptome assembly. Global transcriptome analysis of the hybrids and parental lines showed all possible modes of gene action (additive and non‐additive) in both the hybrid combinations. It was noted that all classes of non‐additive gene action (high (+, positive) and low (−, negative) parent dominance, partial dominance and overdominance) were present in both the analysed hybrids. Of these, approximately 20% and 12% of the genes showed high‐parent dominance in ICPH 2671 and ICPH 2740, respectively. These findings revealed that as reported in several earlier studies, multiple modes of gene actions contributed towards heterosis in pigeonpea (Shen *et al.*, 2017; Swanson‐Wagner *et al.*, 2006).

To further get insight into the altered gene expression changes in hybrids as compared to their parental lines, we analysed hybrid‐MPV DEGs as they are known to be associated with altered gene expressions leading to heterotic phenotype in hybrids (Shen *et al.*, 2017). GO enrichment analysis of hybrid‐MPV DEGs showed enrichment of genes associated with plant growth, stress tolerance, defence and hormones related biological processes. In both the hybrids, transcriptional changes reflected overexpression of genes associated with stress tolerance and plant growth and over‐repression of genes from defence‐related pathways. It has also been observed that 70% of down‐regulated DEGs in ICPH 2671 associated with heterosis are DMGs (methylated‐hybrid‐MPVs DEGs), whereas 67% of down‐regulated DEGs in ICPH 2740 associated with heterosis are DMGs.

Changes in genes associated with carbohydrate metabolism including sugar transporters (*STP1* and *SWEET17*) and nitrogen and sugar metabolism (*BGAL*, *RFS* and *SS*) were up‐regulated suggesting an elevated level of carbohydrate metabolism in hybrids as compared to MPV. This may be linked to increased growth performance in hybrids. It was noted that as there was no methylation observed in these genes, except *SS*, as most of these genes were up‐regulated. Changes in sucrose synthase and other metabolic genes involved in heterosis were also reported earlier in rice hybrids (Counce and Gravois, 2006) and soya bean (Raju *et al.*, 2018). Circadian rhythm plays a crucial role in maintaining key physiological processes in plants and epigenetic alterations in these genes contribute to heterosis by modulating circadian rhythm (Ng *et al.*, 2014). In our study, we found two circadian rhythm associated genes (*CCA1* and *LHY*) that were up‐regulated in hybrids as compared to MPV. However, methylation in *CCA1* was observed while *LHY* was un‐methylated. Epigenetic modifications in *CCA1* leading to changes in circadian rhythm and carbon fixation promoted heterosis in maize and rice (Ko *et al.*, 2016; Song *et al.*, 2010). *CCA1* and *LHY* encode closely related single MYB domain transcription factors in circadian oscillators (Green and Tobin, 2002). Given a competition between plant immunity and plant growth for resource allocation, reduction in basal defence level could be important in generating heterosis (Huot *et al.*, 2014). Our results also showed a reciprocal expression pattern between genes associated with plant growth and defence associated genes (*PR1* and *PR2*). Interestingly, both of these genes showing down‐regulation in hybrids were methylated.

Salicylic acid and auxins were another two important hormones among the defence and stress‐related differentially expressed genes identified in the two hybrids. Given that SA and auxin are key regulators of plant defence responses and are also major controllers of plant structure and growth, altered gene expression in these genes are of potential importance in generating the heterotic phenotype (Busov *et al.*, 2008; Kazan and Manners, 2009). In both the selected hybrids, three auxin‐inducible genes, *SAUR*, *IAA1* and *IAA24,* were overexpressed and methylated as well which might be triggering plant vigour in hybrids. Changes in genes associated with flavonoid biosynthesis pathways also contribute to negative regulation of polar auxin transport, and a small decrease in genes of flavonoid biosynthesis pathway can increase plant growth (Li and Zachgo, 2013). Out of three, two genes (*F3H* and *FL*) of flavonoid biosynthesis pathway shown down‐regulation in both the hybrids as compared to MPV and also were methylated. Two SA pathway‐related genes, *PAL1*, and *PAL17.1,* were shown differential gene expression in hybrids as compared to the MPV; however, the two hybrids have reciprocal expression patter for the two genes. Reports showed that lower SA concentration is associated with increasing leaves and greater cell wall expansion (Miura *et al.*, 2010; Vicente and Plasencia, 2011). In our analysis, *XTH6*, *XTH8*, *BGAL10*, *BXL5* and *BXL7* which are promotor of cell expansion were found to be up‐regulated in both the hybrids. SA regulates ethylene response factors (ERF) which are associated with increased plant growth, activate abiotic stress tolerance and down‐regulate defence‐related genes (Dombrecht *et al.*, 2007; Dubois *et al.*, 2013; Sewelam *et al.*, 2013). Three ERFs (*ERF1A*, *EFR061* and *ERF106*) were up‐regulated, and two among them were methylated in both the hybrids.

### miRNAs play an important role in hybrid performance

miRNAs related to plant growth and defence responses are differentially regulated, affecting the expression pattern of their respective targets resulting in a change in overall metabolism (Zhang *et al.*, 2019). The miR166 family is considered to have several targets, including *ATHD‐ZIP* involved in shoot apical meristem (SAM) and lateral organ development, root initiation and *ARF* genes involved in auxin signalling pathway counterparts to heterosis (Schlereth *et al.*, 2010; Shen *et al.*, 2017). The miR169 family reported being associated with ABA‐responsive transcription factor and *JAZ3* which is known for regulating plant defence response (Ding *et al.*, 2012; Song *et al.*, 2018). The other family, miR171 is known to target transcription factors such as *SCL6* which involves regulation of plant development and have association with biological processes such as circadian rhythm, cell division and cell differentiation (Ma *et al.*, 2014; Grimplet *et al.*, 2016). Growth regulating factors (GRF) is silenced by miR396 by targeting *GRF* genes controlling the regulation in growing and developing tissues (Chandran *et al.*, 2019; Ding *et al.*, 2012)*.*

Previously, the miR408 family is reported to be down‐regulated to target *LAC2* and *SVR7* which leads to promoting root elongation and chloroplast accumulation during photosynthesis, *CALS6* leads to plant growth and stress tolerance (Kuo *et al.*, 2019; Song *et al.*, 2018). The miR164 is predominantly repressed in hybrid indicating an up‐regulation in *NAC1* transcription which promotes auxin signalling for lateral root development (Ding *et al.*, 2012; Fang *et al.*, 2014; Rosas *et al.*, 2017). The up‐regulation of miRNA can cause changes in plant growth, and regulation like auxin‐mediated signalling pathway is governed by *ARF* genes which are targeted by miR167, found up‐regulated in hybrids (Ding *et al.*, 2012; Zhang *et al.*, 2014). miR156 are repressed in hybrids targeting the *SPL* transcription families by interpreting the developmental process by activation of other transcription families (Liu *et al.*, 2017). In our study, miR8724 which is up‐regulated predicted to target F‐box protein in both the hybrids ICPH2043 and ICPH2740, which regulates auxin signalling negatively (Lavy and Estelle, 2016). Identified *miR3476a‐3p* in ICPH2740 regulates *GAM1,* which plays an important role in gibberellin signalling pathways, flower and organ development, and early anther development through aleurone cells is targeted by *miR319c* and *miR319d‐3p* (Tsuji *et al.*, 2006).

## Conclusions

We found that both the hybrids have transcription factors implying increased stress tolerance and suppression of defence‐related genes as major altered process. Although the exact pattern of changes differs between the hybrids, substantial changes in hormone‐regulated genes and metabolic pathways were observed in both the hybrids, accounting for the changes in stress and defence responsive gene expression and possibility for the greater growth of hybrids. Further, epigenetic modifications (DMGs: methylated‐hybrid‐MPV DEGs) in the key genes associated with the identified regulatory pathways associated with heterosis were also observed. We have also identified several common and unique miRNA present in the hybrids, playing an important role through regulation of target genes associated with plant growth, circadian rhythm, plant defence and stress tolerance. Therefore, taken together our results gave an insight into key regulatory networks and genes associated with hybrid vigour. Epigenetic modifications in key genes also found to play important role in hybrids that can alter complex regulatory networks, thus modulating biomass and leading to heterosis in pigeonpea.

## Materials and methods

See also Appendices S1 and S2.

### Plant materials

Three independent biological replicates, each consisting of around 30 pooled seedlings of hybrids, ICPH 2671 and ICPH 2740 and their parents ICPA 2043, ICPA 2047 (CMS line or female parent) and ICPR 2671, ICPR 2740 (restorer line or male parent) were used to construct bisulfite sequencing, transcriptome sequencing and small RNA sequencing.

### Bisulfite sequencing

Genomic DNA was fragmented to 100–300 bp by Sonication followed by DNA‐end repair. DNA fragments were 3′‐dA over hanged and ligated with methylated sequencing adaptors. Bisulfite treatment was given to the adaptor‐ligated DNA fragments using ZYMO EZ DNA Methylation‐Gold kit. After Bisulfite treatment, desalting, size selection, PCR amplification, and again size selection was performed. The qualified libraries were then subjected to bisulfite sequencing.

### Transcriptome sequencing

Total RNAs were isolated using TRIzol reagent (Invitrogen Corp., Carlsbad, CA) and treated with RNase‐free DNase I (New England Biolabs, Ipswich, MA) to remove any contaminating genomic DNA. mRNA extraction was performed using Dynabeads oligo(dT) (Dynal; Invitrogen Corp.). Double‐stranded cDNAs were synthesized using reverse transcriptase (Superscript II; Invitrogen Corp.) and random hexamer primers. The cDNAs were then fragmented by nebulization, and the standard Illumina protocol was followed thereafter to create the mRNA‐seq libraries. For mRNA profiling, *de novo* transcriptome sequencing was performed on the platform of Illumina HiSeq 2000 following the manufacturer's protocol.

### small RNA sequencing

sRNAs were gel‐purified from total RNAs and were subsequently ligated with 3′ and 5′ adapters, followed by reverse transcription using a 3′ reverse transcriptase primer. The cDNAs were then amplified by PCR using primers specific to sRNAs (Mi *et al.*, 2008). After gel purification, the sRNA‐seq libraries were subjected to Illumina sequencing following the manufacturer's protocol. Genomic DNAs were isolated using a commercial kit (DNeasy Plant Maxi Kit; Qiagen Inc., Valencia, CA).

### Identification of differential methylated regions (DMRs)

Sequencing data were filtered, and the low‐quality data were removed. The clean data were mapped to the pigeonpea reference genome (http://cegsb.icrisat.org/gt‐bt/iipg/genomedata.zip). Only the uniquely mapped reads were used for standard analysis and personal bioinformatics analysis. Again, the qualified aligned data (uniquely mapped) was used to get cytosine methylation information at the whole‐genome level. The cytosine methylation information was used for further standard bioinformatics analysis and personalized bioinformatics analysis (see Appendix S2 for details).

### Development of *de‐novo* assembly and identification of DEGs

*De‐novo* assembly of all the six samples was developed based on the standard procedures. DEGs were calculated based on the FPKM value as described in Filloux *et al. *(2014). We used REViGO to cluster the functional categories across the list of significantly enriched functional GO terms (see Appendix S2 for details).

### Processing of small RNA sequencing data

A custom Perl script was first used to remove the 3′ adapter sequences. Next, we compared the trimmed sRNA reads with the NCBI plant tRNA and rRNA databases to remove potentially degraded rRNA and tRNA products from our data sets. We then mapped the remaining trimmed reads to the pigeonpea genome using Bowtie (Langmead *et al.*, 2009), only accepting perfect matches. After mapping, only reads mapped to unique loci were counted for subsequent analyses. A sRNA cluster was defined to contain a minimum of three small RNA reads, and neighbouring sRNA clusters located <200 nt apart were merged together.

### Data processing, bioinformatics analyses and data availability

See Appendices S1 and S2 for details about data processing and bioinformatics analyses. All sequencing data generated have been deposited to National Center for Biotechnology Information (NCBI) Sequence Read Archive (SRA) database under the BioProject ID: PRJNA549058.

## Conflict of interests

The author(s) declare that they have no competing interests.

## Authors' contributions

RKV conceived the idea and supervised the study. PS performed most of the analysis. PS, VKS, RKS and RKV interpreted the results and wrote the manuscript. AC, YL, TM, XL and XX contributed to data generation. PS, SK, VG, ML and AWK carried out statistical analysis. KBS and CVS provide genetic materials. KDK, SJ, WP, EN, IRS and ML contributed to analysis and interpretation of results. All authors read and approved the final manuscript.

## Supporting information


**Figure S1** Cumulative distribution of effective sequencing depth in Cytosine of both the hybrids and their parental lines.
**Figure S2** The proportion of different methyl‐cytosine patterns of both the hybrids and their parental lines.
**Figure S3** Canonical DNA methylation profiles of ICPH 2671 and its parental lines.
**Figure S4** Canonical DNA methylation profiles of ICPH 2740 and its parental lines.
**Figure S5** Heat maps show distinct methylation and CpGs (CG, CHG, CHH) density patterns for both the hybrid combinations across the genome and at different regulatory units.
**Figure S6** Methyl‐cytosine density distribution of ICPH 2671 and its parental.
**Figure S7** Methyl‐cytosine density distribution of ICPH 2740 at different CG.
**Figure S8** Comparative analysis of per cent methylation contributed from parental lines in hybrid (a) ICPH 2671 and (b) ICPH 2740.
**Figure S9** Distribution of DMRs of different cytosine contexts in different regions for (a) ICPH 2671 and (b) ICPH 2740 hybrid combinations.
**Figure S10** Class distribution analysis of filtered reads in hybrids and parental lines showed that 21 and 24 nucleotide classes were the most abundant groups in (a) ICPH 2671 (b) and its parental lines.
**Figure S11** More enrichment of sRNA was observed in intergenic regions followed by non‐TE related genes and TE‐related genes in both the hybrids and their parental lines.
**Figure S12** NR Classification of Unigenes (A) The E‐ value distribution of the result of NR annotation. (B) The similarity distribution of the result of NR annotation. (C) The species distribution of the result of NR annotation.
**Figure S13** COG function classification of Unigenes in All‐Unigenes.
**Figure S14** GO classification analysis of Unigenes.
**Figure S15** Number of DEGs identified between different combinations of hybrids and parental lines.
**Figure S16** Expression levels of genes (DEGs and non‐DEGs) between different combinations of hybrid (ICPH 2671) and its parental lines (ICPA 2043 and ICPR 2671).
**Figure S17** Expression levels of genes (DEGs and non‐DEGs) between different combinations of hybrid (ICPH 2740) and its parental lines (ICPA 2047 and ICPR 2740).
**Figure S18** Expression levels of genes (DEGs and non DEGs) between different hybrids (ICPH 2671 and ICPH 2740).
**Figure S19** Classification of gene action in (a) ICPH 2671 and (b) ICPH 2740 hybrid combination based on the genome‐wide transcriptome data.
**Figure S20** Global crosstalk between genome‐wide epigenetic regulation, miRNA, sRNA and gene expression in realizing pigeonpea heterosis.
**Table S1** Heterotic phenotypes of 15 and 30 days‐old pigeonpea F_1_ hybrids relative to their parents.
**Table S2** DNA methylation reads generated and alignment of parental lines and hybrids.
**Table S3** Pairwise comparison of DMRs among parents and their hybrids.
**Table S4** List of common DMRs among parental lines and hybrid (*Datasets*).
**Table S5** List of DMRs among parental lines and hybrid within the gene elements.
**Table S6** Classification of DMRs among parental lines and hybrids.
**Table S7** Statistics of small RNA sequencing data production.
**Table S8** Statistics of small RNA distribution identified in genic and flanking (up and downstream).
**Table S9** Association of DMRs and sRNA among ICPH 2671 and its parental lines.
**Table S10** Association of DMRs and sRNA among ICPH 2740 and its parental lines.
**Table S11** Comparative analysis of small RNA (sRNA) and DNA methylation in the different CG context.
**Table S12** List of differentially expressed miRNA and their target expression in ICPH 2671 and ICPH 2740 hybrid combinations (*Dataset*).
**Table S13** Statistics of RNA sequencing data production.
**Table S14** Details of the development of contigs of parental lines and hybrids.
**Table S15** Details of the development of *de‐novo* assemblies of parental lines and hybrids.
**Table S16** Details of annotations of unigenes against different databases.
**Table S17** List of differentially expressed genes (DEGs) between parental lines and hybrids.
**Table S18** Statistical analysis and classification of genes based on gene action.
**Table S19** Identification of DEGs associated DMRs present in genic and flanking (2 kb up and downstream) regions.
**Table S20** REVIGO amalgamated GO biological process associated with the hybrid‐MPV DEGs for ICPH 2671 and ICPH 2740 hybrid combinations.
**Appendix S1** Supplementary text (SI text).
**Appendix S2** Materials and Methods.
