# Supplementary material for: Genome‐wide analysis of epigenetic and transcriptional changes associated with heterosis in pigeonpea
Source: Plant Biotechnol J. 2020 Feb 3;18(8):1697–710. doi: 10.1111/pbi.13333 (PMC7336283; doi:10.1111/pbi.13333)
Supplement: Supplementary file 1 — Figure S1 Cumulative distribution of effective sequencing depth in Cytosine of both the hybrids and their parental lines. Figure S2 The proportion of different methyl‐cytosine patterns of both the hybrids and their parental lines. Figure S3 Canonical DNA methylation profiles of ICPH 2671 and its parental lines. Figure S4 Canonical DNA methylation profiles of ICPH 2740 and its parental lines. Figure S5 Heat maps show distinct methylation and CpGs (CG, CHG, CHH) density patterns for both the hybrid combinations across the genome and at different regulatory units. Figure S6 Methyl‐cytosine density distribution of ICPH 2671 and its parental. Figure S7 Methyl‐cytosine density distribution of ICPH 2740 at different CG. Figure S8 Comparative analysis of per cent methylation contributed from parental lines in hybrid (a) ICPH 2671 and (b) ICPH 2740. Figure S9 Distribution of DMRs of different cytosine contexts in different regions for (a) ICPH 2671 and (b) ICPH 2740 hybrid combinations. Figure S10 Class distribution analysis of filtered reads in hybrids and parental lines showed that 21 and 24 nucleotide classes were the most abundant groups in (a) ICPH 2671 (b) and its parental lines. Figure S11 More enrichment of sRNA was observed in intergenic regions followed by non‐TE related genes and TE‐related genes in both the hybrids and their parental lines. Figure S12 NR Classification of Unigenes (A) The E‐ value distribution of the result of NR annotation. (B) The similarity distribution of the result of NR annotation. (C) The species distribution of the result of NR annotation. Figure S13 COG function classification of Unigenes in All‐Unigenes. Figure S14 GO classification analysis of Unigenes. Figure S15 Number of DEGs identified between different combinations of hybrids and parental lines. Figure S16 Expression levels of genes (DEGs and non‐DEGs) between different combinations of hybrid (ICPH 2671) and its parental lines (ICPA 2043 and ICPR 2671). Figure S17 Expression level [file PBI-18-1697-s002.docx]

**Supplementary Information**

**Genome-wide analysis of epigenetic and transcriptional changes associated with heterosis in pigeonpea**

Pallavi Sinha^1,†^, Vikas K Singh^1,†^, Rachit K Saxena^1,†^, Sandip Kale^1^, Yuqi Li^2^, Vanika Garg^1^, Tang Meifang^2^, Aamir W Khan^1^, Kyung Do Kim^3,4^, Annapurna Chitikineni^1^, KB Saxena^1^, CV Sameer Kumar^1^, Xin Liu^2^, Xun Xu^2^, Scott Jackson^3^, Wayne Powell^5^, Eviatar Nevo^6^, Iain R. Searle^7^, Mukesh Lodha^8^, Rajeev K Varshney^1,^*

^1^Center of Excellence in Genomics & Systems Biology, International Crops Research Institute for the Semi-Arid Tropics, Patancheru, 502324, Telangana State, India

^2^BGI-Shenzhen, Yantian District, Shenzhen, 518083, China

^3^University of Georgia, Athens, USA

^4^LG Science Park, 30 Magokjungang 10-ro, Gangseo-gu, Seoul, 07796, Republic of Korea

^5^Scotland's Rural College (SRUC), Edinburgh, UK

^6^Institute of Evolution, University of Haifa, Mount Carmel, Haifa, 3498838, Israel

^7^Department of Molecular and Biomedical Sciences, School of Biological Sciences, The University of Adelaide, Adelaide, SA, 5005, Australia

^8^Centre for Cellular and Molecular Biology (CSIR), Habsiguda, 500007, Telangana State, India

^†^Authors contributed equally to this work

**^*^Author for Correspondence**

Rajeev K Varshney

Center of Excellence in Genomics & Systems Biology

International Crops Research Institute for the Semi-Arid Tropics (ICRISAT)

Patancheru - 502 324, India

Telephone: 91-40-30713305;

Fax: 91-40-30713074

E-mail: r.k.varshney@cgiar.org

**Appendix S1**

**Hybrids and their phenotypic performance over parents**

To study the possible mechanism underlying heterosis, we selected two leading pigeonpea hybrids and the parental lines based on previous yield data. ICPH 2671 is a CMS-based medium-duration pigeonpea hybrid and yielded 41.6% more than the control variety Maruti. This hybrid was released as ‘Pushkal’ (Pravardhan Seeds, India) and ‘RV ICPH 2671’ ([Maharana Pratap University of Agriculture and Technology, Udaipur, India)](https://www.researchgate.net/institution/Maharana_Pratap_University_of_Agriculture_and_Technology). In All-India Co-ordinated (IHT & AHT) trials conducted in 2007, the yield of ICPH 2671 was 31% higher in the central zone and 62% higher in the south zone over the control variety Maruti. ICPH 2740 another commercially released hybrid, released under the name Mannem Konda with a yield potential of 3.5 tons per ha it registered a 40% yield increase over the local cultivars. Consistent with the previous reports both commercial *hybrids (*ICPH 2671 and ICPH 2740) of pigeonpea showed a higher level of heterosis in terms of leaf shape, root length and plant height in comparison to their parental lines (ICPA 2043 and ICPR 2671 for ICPH 2671 and ICPA 2047 and ICPR 2740 for ICPH 2740). The phenomenon of heterosis was clearly visible after 15 days of sowing (**Table S1 and Figure 2a-e**).

**Bi-sulfite sequencing of hybrids and their parental lines**

MethylC-seq libraries of both the hybrids and their parental lines were prepared and sequenced on an Illumina HiSeq 2000. A total of 543.2 and 589.9 million paired-end (2 x100 PE) reads were generated for ICPH 2671 and their parental lines, and ICPH 2740 and their parental lines, respectively (**Table S2**). The sequencing reads from each library were mapped to the pigeonpea reference genome using an inbuilt algorithm to determine the frequency of reads matching each genomic position (**see Supplementary Materials and Methods for details**). Mapping of raw reads to the reference genome revealed an average read depth of 25.3 X (91.4 % reads mapped), 24.4 X (91.6 % reads mapped), and 24.5 X (92.7 % reads mapped) for ICPA 2043, ICPH 2671 and ICPR 2671, respectively. For another hybrid combination average read depth of 25.2 X (91.8 % reads mapped), 25.0 X (92.1 % reads mapped) and 30.5 X (92.5 % reads mapped) for ICPH 2740, ICPA 2047 and ICPR 2740, respectively were observed after mapping (**Table S2**).

**Methylation level in *hybrids and their parental lines***

There are three possible methylated cytosine patterns, CG, CHG, and CHH (where H=A, T or C)***.*** Cumulative distribution of effective sequencing depth in cytosine of both the hybrids and their parental lines are presented in **Figure S1 and Table S3**. CG methylation was highest, followed by CHG and CHH methylation in both of the hybrids, ICPH 2671 and ICPH 2740 hybrids and their parental lines ICPA2043/ICPR 2671 and ICPA 2047/ICPR 2740, respectively (**Figure S2**) Methylation levels were determined by dividing the number of non-converted reads covering each methylcytosine (mC) by the total reads covering that cytosine, which was also equal to the mC/C ratio at each reference cytosine. The average methylation levels in both the hybrids ICPH 2671 and ICPH 2740 was found significantly higher (p<0.001), relative to their parents. Further DNA methylation patterns were studied for different genomic features and it was found that CG and CHG context showed comparatively higher levels of DNA methylation across different regulatory features for both the hybrid combinations (**Figures S3-S4**) as compared to CHH. Canonical DNA methylation profiles of both the hybrids and their parental lines were studied in 6 different features (genome, promoter, genic, exon, intron and intergenic) and it was found that higher level of methylation reported in intergenic region followed by genome and promoter regions (**Figures S3-S4**).

To understand the DNA methylation patterns of all cytosine contexts across the transcriptional unit at a whole-genome level, each transcriptional unit was studied at distinct functional elements level. It was observed that for each cytosine context, mean methylation level was higher at upstream (including TSS region) and downstream regions. However, methylation level at internal intronic and exonic regions was lower for both hybrid combinations (**Figure S5**). The methyl-cytosine density of each chromosome was also studied, and large variation was observed for each cytosine context throughout each chromosome for both the hybrid combinations (**Figure S6-S7**). Comparative analysis between hybrids and their parental lines was analyzed to gain more insight into the methylation level. Each hybrid was compared in pairwise combinations for all the three-cytosine contexts (**Table S4**). Both the hybrids possess a higher level of methylation in all of the cytosine context in comparison to the CMS (ICPA 2043, ICPA 2047) and restorer parents (ICPR 2671, ICPH 2740). Comparative analysis of percent methylation contributed from parental lines in hybrid revealed 85.56% (ICPH 2671) and 88.87% (ICPH 2740) were the positions where methylation was contributed by both the parents. It was found that 3.86% and 5.63% of methylation was uniquely contributed from ICPA 2043 and ICPR 2671 respectively in the hybrid ICPH 2671 (**Figure S8a**). Similarly, ICPA 2047 and ICPR 2740 contributed methylation of 2.01% and 2.23% uniquely to the hybrid ICPH 2740 (**Figure S8b**). Interestingly, it was noted that at 2.69% (ICPH 2671) and 1.50% (ICPH 2740) position, there was no methylation present in hybrids, but either of the parents was methylated.

***Identification of differentially methylated regions (DMRs)***

To investigate the differential methylation in hybrids and their parental lines, a sliding-window approach was followed to identify differentially methylated regions (DMRs) which contained at least five CG, CHG, CHH sites and where methylation level of two different samples was significantly different (p-value ≤ 0.001). Using these criteria, 52,281 DMRs between ICPA 2043/ICPH 2671, 41,351 DMRs between ICPR 2671/ICPH 2671 and 48,461 DMRs between ICPA 2043/ICPR 2671 could be identified (**Figure S9**). To identify the common DMRs between parents and hybrid, all three samples were analyzed together (**Table S4**). As a result, 13,987 DMRs were identified. Similarly, 42,011, 41,472 and 55,583 DMRs were identified between ICPA 2047/ICPH 2740, ICPR 2740/ICPH 2740 and ICPA 2047/ICPR 2740 respectively. Combined analysis of both the parents and hybrids revealed 15,132 common DMRs among ICPH 2671 hybrid combination (**Table S4**). Among the DMRs between ICPA 2043/ICPR 2671 (13,987) 3,587 had higher methylation levels in ICPR 2671 and 10,400 in ICPA 2043. In the case of ICPA 2047/ICPR 2740 (15,132), 2,620 DMRs had higher methylation levels in ICPR 2740 and 12,512 in ICPA 2047 (**Figure S9 and Table S4**). To account for these differences in the predicted additive methylation level in hybrids, we estimated the mean methylation level by calculating mid-parental values (MPV) of methylation. The mid-parent values (MPVs) were calculated to identify interactive and non-interactive DMRs. A total of 96.59% (13,511; ICPH 2671 and its parental lines) and 97.38 % (14736; ICPH 2740 and its parental lines) of DMRs were found significantly different from MPVs, suggesting that there is substantial methylation interaction (interactive DMRs) at these regions. However, a very less percentage of non-interaction (NI) DMRs 3.40% (476 DMRs in ICPH 2671 and its parental lines) and 2.60% (394 DMRs in ICPH 2740 and its parental lines) were observed in both the combinations (**Table S4**). While analyzing the distribution of DMRs, it was found that in ICPH 2671 combination, DMRs were more likely to be located near 2 kb upstream (45.92 to 46.98%) and 2 kb downstream (44.27 to 45.40%) regions of the gene. However, within the gene, less percentage (7.73 to 9.80%) of DMRs were observed. For hybrid combination ICPH 2740, a similar pattern was observed with higher percentage of DMRs in 2 kb upstream (46.14 to 46.79%) and 2 kb downstream (45.41 to 45.74%) regions of the gene followed by lesser percentage of DMRs within gene (7.46 to 8.34%) (**Table S5**). The DMRs within the gene and within 2 kb flanking regions were termed as DMR-associated genes. Overall, 26.99% and 24.58% DMR-associated genes were found in ICPH 2671 and its parental lines and ICPH 2740 and its parental lines, respectively (**Table S5**).

**sRNA profiling and their association with DNA methylation**

***sRNA profiling of hybrids and parental lines***

To investigate the relationship between DNA methylation and sRNAs in pigeonpea, a correlation between small RNA (sRNA) abundance and DNA methylation was analyzed. To perform genome-wide sRNAs profiling, small RNA libraries for ICPH 2671, ICPH 2740 and their parental lines were sequenced. A total of ~143 million reads (49 bp per read) were obtained from the six sequenced libraries (**Table S7**). Pre-processing (removal of low-quality reads, adapter/primer trimming and reads smaller than 18 nucleotides were discarded) of data resulted in ~142 million clean reads, of which ~120 million reads were mapped to the pigeonpea genome. After removing reads mapped to rRNA, tRNA, small nuclear RNA and nucleolar RNA, ~78 million sRNA reads were finally obtained. The number of unique sRNAs showed similar counts (±5 million) for all the six genotypes. Further, class distribution analysis of filtered reads in hybrids and parental lines showed that 21 and 24 nucleotide classes were the most abundant groups for both the hybrid combinations (**Figure S10a-b).** In both hybrid combinations, the non-TE genes showed more enrichment of sRNA than TE-related genes and a large number of sRNA reads mapped to intergenic regions (**Figure S11**). sRNA was in higher frequency in non-TE-related regions, in ICPH 2671 as compared to mid parental value (MPV) whereas, in ICPH 2740, sRNA frequency was lower in comparison to the MPV. The integration of genomic coordinates of sRNAs with the pigeonpea genome annotation revealed ~12% of sRNAs was originated from the genic and flanking sequences for both the hybrids (**Table S8**).

***Relationship between sRNA and DNA methylation***

We analyzed our data to investigate the relationship between sRNAs and DNA methylation. It was found that DNA methylation levels were significantly higher in the regions with sRNAs than those without sRNA in both the hybrid combinations (**Figures 4a-4c**). Moreover, in the regions with sRNAs, DNA methylation levels in all three cytosine contexts were *at par* or slightly higher in hybrids than their respective parents (**Figure 4d, Table S11**). To characterize how differences in DNA methylation in the parental lines affected DNA methylation in hybrids, we grouped the cytosines into four categories based on the methylation levels of the parental lines (i) positions highly methylated in CMS line than restorer line (ICPA 2043 > ICPR 2671; ICPA 2047 > ICPR 2740), (ii) positions highly methylated in restorer line than CMS line (ICPR 2671 < ICPA 2043; ICPR 2740 < ICPA 2047), (iii) positions where methylation was detected but levels were equal in CMS and restorer lines (ICPA 2043 = ICPR 2671 > 0; ICPA 2047 = ICPR 2740 > 0), and (iv) positions lacking detectable methylation in both CMS and restorer lines (ICPA 2043 = ICPR 2671 = 0; ICPA 2047 = ICPR 2740 = 0) (**Figures 4e-4f**). Analysis indicated that regions covered by sRNAs that were differentially methylated in the parents contributed 72.9% and 69.0% of the increased methylation in ICPH 2671 and ICPH 2740, respectively (**Figures 4e-4f** ). This indicates the mobility of sRNAs that mediate epigenetic regulation renders them promising candidates for modulating transgressive phenotypes in hybrids.

**Transcriptome analysis of hybrids and their parental lines**

***De-novo assembly development***

To investigate whether the observed changes in DNA methylation lead to altered gene expression in hybrids, we measured the transcriptome of hybrids and their parents by mRNA sequencing. A total of 260.28 million paired-end reads were obtained from the libraries of six genotypes (**Table S13**). After stringent quality checking and data cleaning, approximately 242.93 million high-quality paired-end reads have been achieved with 97.33 to 97.47 % reads with Q20 bases (base quality more than 20). The GC content (ratio of guanine and cytosine) in the genotypes ranged from 44.97 % to 46.54 %. It is important to note that, because the pigeonpea reference genome assembly is not of the gold standard, *de novo* assembly of hybrids and parental lines was generated. To recreate the original sequence and unknown transcripts/genes, *de novo* assemblies of hybrids and their parental lines obtained from mRNA sequencing data were assembled (**Table S14 and S15**). Further, for the development of the *de-novo* assembly filtered reads were assembled using Trinity de-novo assembler (specifically for use with next-generation short-read sequences) into full-length transcripts of the six genotypes. Based on the high-quality reads 53, 811 (ICPH 2671) to 80, 122 (ICPR 2740) contigs, with a mean average length of 505 (ICPH 2740) to 600 bp (ICPA 2043) were assembled (**Table S14-15**). To obtain unigenes, the pair-end reads were realigned to contigs and gained the sequence, which is not being extended on either end were defined as ‘unigenes’. Finally, the de-novo assembly of individual six genotypes was constructed, and the number of unigenes ranged from 32116 in ICPA 2043 to 53776 in ICPR 2740 with an average length of 1634 bp (ICPH 2671) to 1812 bp (ICPH 2740). The N50 ranged from 1634 in ICPH 2671 to 1812 in ICPG 2740 (**Table S14-15**).

Further, TIGR Gene Indices clustering tools (TGICL; Pertea, 2003) was used to assemble all the unigenes from all six samples to form a single set of 63,825 non-redundant unigenes with N50 (**Figure S12**) which were further annotated by performing BLASTX (Basic Local Alignment Search Tool) search against non-redundant protein (Nr; **Figure S12**), non-redundant nucleotide (NT), Swiss-Prot protein database, Kyoto Encyclopedia of Genes and Genomes Pathway database (KEGG), Clusters of Orthologous Groups (COG; **Figure S13**) database Gene Ontology (GO; **Figure S13**) database with e-value cut-off of (E-value ≤ 1.0E^-5^). As a result, 53,996 genes were annotated with these databases (**Table S16**). The unigenes were further subjected to differential gene expression analysis by using Fragments per kb per million reads (FPKM) method for calculating expression values. GO analysis of unigenes predicted biological, molecular and cellular functions (**Figure S14**)

***Genetic dissection to understand the mode of gene action***

Setting FPKM >2, log fold >1 and FDR < .001 a total of 7, 223 (ICPH 2671 combination) and 12,558 (ICPH 2740 combination) DEGs were further investigated to determine their modes of gene action (**Figures S19a-19b**). Genes in the hybrid having the expression levels significantly not deviated and deviated from MPV were designated as additive and non-additively expressed genes respectively. In ICPH 2671 triad, 10.69 % and in ICPH 2740 combination 4.93 % showed an expression pattern that was not distinguishable from MPV exhibiting an additive expression pattern. Additionally, 89.31 % (6451 of 7223) in ICPH 2671 combination and 95.07 % in ICPH 2740 triad (11639 of 12558) DEGs showed a non-additive expression pattern. The non-additive DEGs from the cross were further classified into different groups (**Table S18**). In the case of ICPH 2671, 1427 (19.73 %) and 1291 (17.87 %) genes exhibited positive and negative over dominance, respectively. However, many genes showed partial dominance (3730 genes; 51.64 %). Similarly, for the hybrid combination ICPH 2740, 1519 (12.09 %) and 2344 (18.67 %) genes exhibited positive and negative over dominance, respectively. More than 64 % (8065) genes showed partial dominance. These results demonstrated that many genes exhibited non-additive expression patterns in the two hybrid combinations.

**Appendix S2: Materials and Methods**

**Plant materials**

To study the possible mechanism underlying heterosis, we selected two leading pigeonpea hybrids and the parental lines based on previous yield data. ICPH 2671 (crossed between ICPA 2043; CMS line and ICPR 2671; restorer line) is a CMS-based medium-duration pigeonpea hybrid and yielded 41.6% more than the control variety Maruti. In All-India Co-ordinated (Initial Hybrid Trial & Advanced Hybrid Trial) trials conducted in 2007, the yield of ICPH 2671 (crossed between ICPA 2047; CMS line and ICPR 2740; restorer line) was 31% higher in the central zone and 62% higher in the south zone over the control variety Maruti. ICPH 2740, is another commercially released hybrid, used in the present study which is released under the name Mannem Konda with a yield potential of 3.5 tons per ha it registered a 40% yield increase over the local cultivars. Pure seeds of hybrids (F1 seeds developed trough hand crossing in controlled conditions) and parental lines (pure inbred lines generated by selfing for several generations in controlled conditions) were obtained from Pigeonpea Breeding Division of ICRISAT, Hyderabad. The hybrid purity of the F1s was tested with the gene-based/linked markers specific with the male and female parents of the hybrids before initiating the sequencing of the samples. Cytoplasm male sterility-based marker **(Sinha et al. 2015**) was utilized to detect the presence of A4 cytoplasm of female parent and restorer gene (Rf) based marker of male parent (**Saxena et al. 2017**) was used to check the F1s heterozygosity for Rf allele.

**The pipeline of bioinformatics analysis**

Sequencing data was filtered, and the low-quality data was removed. The clean data were mapped to the pigeonpea reference genome (http://www.icrisat.org/gt-bt/iipg/genomedata.zip). Only the uniquely mapped reads were used for standard analysis and personal bioinformatics analysis. Again, the qualified aligned data (uniquely mapped) was used to get cytosine methylation information at whole-genome level. The cytosine methylation information was used for further standard bioinformatics analysis and personalized bioinformatics analysis.

**Analysis of methylation data**

*Data filtering*

Data filtering includes removing adaptor sequences, contamination and low-quality
reads from raw reads using in-house programs. The low-quality reads
include three types, and the read which accord with one of them was removed:

(1) contain an adaptor sequence;

(2) N base number over 10%; and

(3) the number of bases whose quality less than 20 over 10% were trimmed.

*Reads Alignment*

Observed cytosine on the forward read of each read pair was in silico replaced by thymine and observed guanines on the reverse read of each read pair were in silico replaced by adenines. The “alignment form” reads were then mapped to the “alignment form” reference genome by BSMAP. Every hit with a single placement with minimum numbers of mismatches and a clear strand assignment was defined as unambiguous alignment (uniquely mapped reads) and was used for methyl-cytosine ascertainment. Only the uniquely mapped reads were used to estimate the copy numbers of the local region.

We used a custom Perl script to compute the average methylation pattern of a set of loci. For each locus, the script creates 50 bins: 20 bins of 100 bp each for the 2 kb upstream and 2 kb downstream of the locus, and ten bins of an equal length corresponding to the locus accession. The methylation level of each bin of a given locus was computed. The average methylation level for each bin in all loci of the set was plotted.

*Statistics of effective coverage*

Effective coverage analysis is performed on three different levels: chromosome, gene
region and genomic features. One cytosine was identified as effectively covered
cytosine when the cytosine’s effective sequencing depth is no less than 1. Effective
coverage was determined by dividing the number of effectively covered cytosine by
the total cytosine in the corresponding region.

$$Effective coverage of one region=100 \times\frac{Effectively covered cytosines}{Total cytosines}$$

*2.3.4. Methylation level*

Methylation level was determined by dividing the number of reads covering each mC
by the total reads covering that cytosine, which was also equal the mC/C ratio at each
reference cytosine. The function is showed as following:

$$Methylation level=100 \times\frac{Reads which covered methyl cytosines}{Effective sequencing reads}$$

*Average methylation level of different regions*
The average methylation level is performed on three different levels: chromosome, gene region and genomic feature. The function of the average methylation level is showed as following:

$$Average methylation level=100 \times\frac{\sum reads which covered methyl cytosines}{\sum effective sequencing reads}$$

*Proportion in Total Methyl-cytosine of CG, CHG, and CHH*

Proportion of different types of methyl-cytosine varies with species, and there is a specific methylation profile under conditions concerning time, space and physiological changes. So, the number of each methyl-cytosine pattern and its proportion in total methyl-cytosine could reveal methylome of pigeonpea.

$$Proportion of mCHG=100 \times\frac{Number of mCHG}{Number of total mC}$$

**Identification of differentially methylated regions (DMRs)**

Sequencing data was filtered, and the low-quality data was removed. The clean data were mapped to the pigeonpea reference genome (**http://cegsb.icrisat.org/gt-bt/iipg/genomedata.zip**). Only the uniquely mapped reads were used for standard analysis and personal bioinformatics analysis. Again, the qualified aligned data (uniquely mapped) was used to get cytosine methylation information at the whole-genome level. The cytosine methylation information was used for further standard bioinformatics analysis and personalized bioinformatics analysis.

Trimming was performed using the paired-end mode, and three types of low-quality reads (1) contain an adaptor sequence; (2) N base number over 10%; and (3) the number of bases whose quality less than 20 over 10% were trimmed. Observed cytosine on the forward read of each read pair was in silico replaced by thymine and observed guanines on the reverse read of each read pair were in silico replaced by adenines. The “alignment form” reads were then mapped to the “alignment form” reference genome by BSMAP. Every hit with a single placement with minimum numbers of mismatches and a clear strand assignment was defined as unambiguous alignment (uniquely mapped reads) and was used for methyl-cytosine ascertainment. Only the uniquely mapped reads were used to estimate the copy numbers of the local region.

We used a custom Perl script to compute the average methylation pattern of a set of loci. For each locus, the script creates 50 bins: 20 bins of 100 bp each for the 2 kb upstream and 2 kb downstream of the locus, and ten bins of an equal length corresponding to the locus accession. The methylation level of each bin of a given locus was computed. The average methylation level for each bin in all loci of the set was plotted. To identify genomic regions with significant differences in methylation between two lines (differentially methylated regions; DMRs), we merged the replicate data together and performed a Wilcoxon rank test. After we merged the replicate data for each line, we first tested whether each cytosine was differentially methylated in the two lines. To do so, we measured the methylation levels in these two lines in a sliding-window approach, which contained at least five CG（CHG, CHH) sites and its methylation levels of two different samples were different. A Wilcoxon test was ran on the methylation values for the windows of the two lines, calculating the p values and then performing an FDR correction. Cytosines with corrected p values (< 0.05) were identified as differentially methylated. After this, concatenated differentially methylated cytosines with at least 10 informative adjacent cytosines and average methylation differences of at least 0.1 for CG and CHG contexts and at least 0.05 for CHH contexts were identified as differentially methylated regions.

***De-novo* assembly and analysis of Illumina reads**

The samples were assembled with SOAPdenovo (**Li et al. 2010**) separately. The reads were first combined to form longer fragments, i.e., contigs. The reads were then mapped back to the contigs, and the paired-end reads and contigs from the same transcript were assembled to form a longer sequence, with N for unknown sequences (i.e., scaffolds). Paired-end reads were again used for gap filling of the scaffolds to obtain unigenes with the least Ns that could not be extended on either end. For future analyses, the unigenes from the six samples were assembled again to acquire non-redundant unigenes (All-Unigenes) that were if possible. All-Unigenes assembled from the three samples were compared with the NCBI non-redundant (NR) protein database using blastx v2.2.14 (**Altschul et al. 1997**) with an E-value cut-off of 1e-5. Based on the results of the protein database annotation, Blast2GO (**Conesa et al. 2005**) was employed to obtain the functional classification of the unigenes based on GO terms. WEGO software (**Ye et al. 2006**) was used to perform the GO functional classification for all the unigenes and to understand the distribution of the gene functions of this species at the macro level. The KEGG database (V56.0, Oct. 1, 2010) (**Kanehisa et al. 2008**) was used to annotate the pathway of these unigenes.

***Identification of differentially expressed genes (DEGs)***

DEGs were calculated based on the FPKM value as described in **Filloux et al. (2014).** The normalization of annotated unigenes was performed to obtain the expression values in FPKM (Fragments Per Kilobase of transcript per Million mapped reads). The FPKM value is calculated only with uniquely mapped reads to the annotated unigenes with the below formula

$$FPKM=\frac{{10}^{6}\times C}{N L /{10}^{3}}$$

In the above-mentioned formula, C is the number of reads that uniquely aligned to a unigene, N to be total number of reads that uniquely aligned to all unigenes, and L is the base number in the CDS of specific Unigene. Only the significant unigene with corrected p-value of FDR <= 0.001 is taken for the identification of differentially expressed genes (DEGs) in the comparison of hybrids and Mid Parent Value (MPV = Parent 1 + Parent 2 /2). DEGs are considered only by Logarithmic transformation while comparing the samples to find upregulated and downregulated genes with at least one-fold change difference.

**References:**

1. Sinha, P., Saxena, K.B., Saxena, R.K., Singh, V.K., Suryanarayana, V., Sameer Kumar, C.V., Katta, M.A.V.S., et al. (2015) Association of *nad7a* gene with cytoplasmic male sterility in pigeonpea. *Plant Genome,* **8**:2
2. Saxena, R.K., Patel, K., Sameer Kumar, C.V., Tyagi, K., Saxena, K.B., Varshney, R.K. (2018) Molecular mapping and inheritance of restoration of fertility (*Rf*) in A4 hybrid system in pigeonpea (*Cajanus cajan* (L.) Millsp.). *Theor. Appl. Genet.,* **131,** 1605–1614
3. Li, R., Zhu, H., Ruan, J., Qian, W., Fang, X., Shi, Z., Li, Y., et al (2010) De novo assembly of human genomes with massively parallel short read sequencing. *Genome Res*., **20**, 265-272.
4. Altschul, S.F., Madden, T.L., Schaffer, A.A., Zhang, J., Zhang, Z., Miller, W., Lipman, D.J. (1997) Gapped BLAST and PSI-BLAST: a new generation of protein database search programs. *Nucleic Acids Res*., **25**, 3389-3402.
5. Conesa, A., Gotz, S., Garcia-Gomez, J.M., Terol, J., Talon, M., Robles, M (2005) Blast2GO: a universal tool for annotation, visualization and analysis in functional genomics research. *Bioinformatics,* **21**, 3674-3676
6. Ye, J., Fang, L., Zheng, H., Zhang, Y., Chen, J., Zhang, Z., Wang, J., et al. (2006) (2006) WEGO: a web tool for plotting GO annotations. *Nucleic Acids Res*., **34**, W293-W297.
7. Kanehisa, M., Araki, M., Goto, S., Hattori, M., Hirakawa, M., Itoh, M., Katayama, T., et al (2008) KEGG for linking genomes to life and the environment. *Nucleic Acids Res.,* **36**, D480-D484.
8. Filloux, C., Cédric, M., Romain, P., Lionel, F., Christophe, K., Dominique, R, Daniel, P. (2014) An integrative method to normalize RNA-Seq data*. BMC Bioinformatics*, **15**, 188.

***Supplementary Figures***


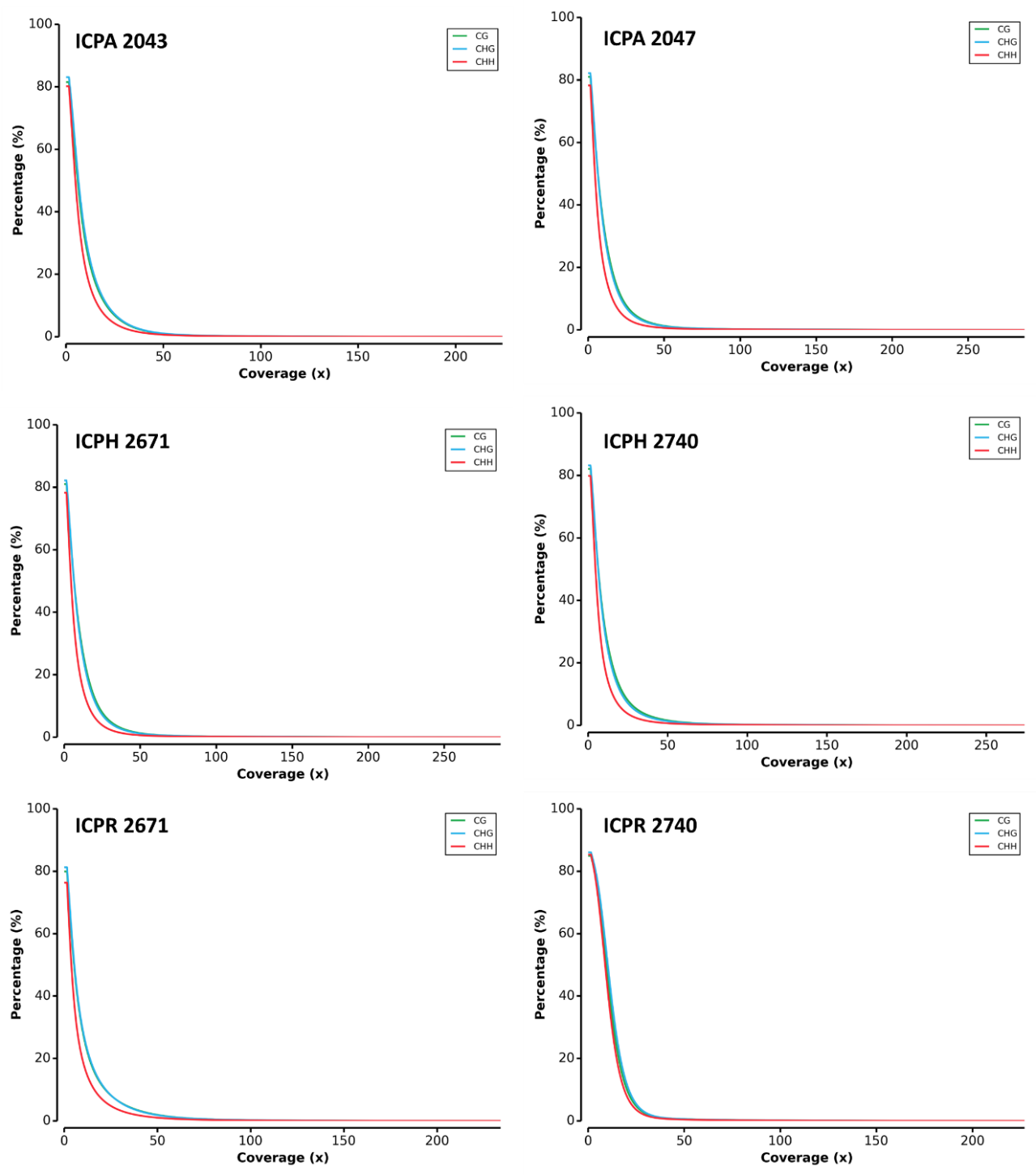


**Figure S1.** Cumulative distribution of effective sequencing depth in Cytosine of both the hybrids and their parental lines. The horizontal axis represents the effective sequencing depth of C while the vertical axis represents the percentage of each kind of C at a certain sequencing depth.


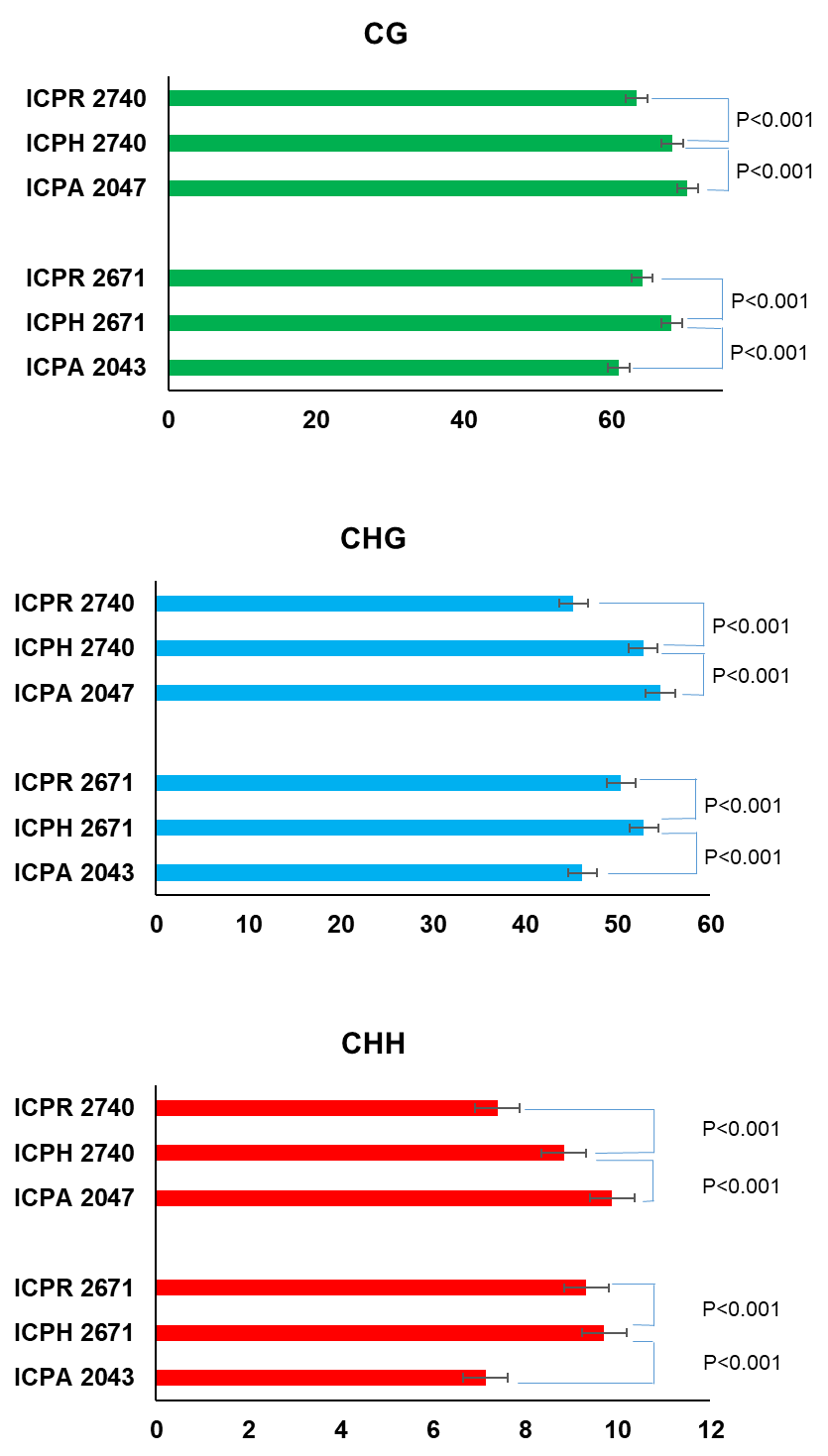


**Figure S2.** The proportion of different methyl-cytosine patterns of both the hybrids and their parental lines.


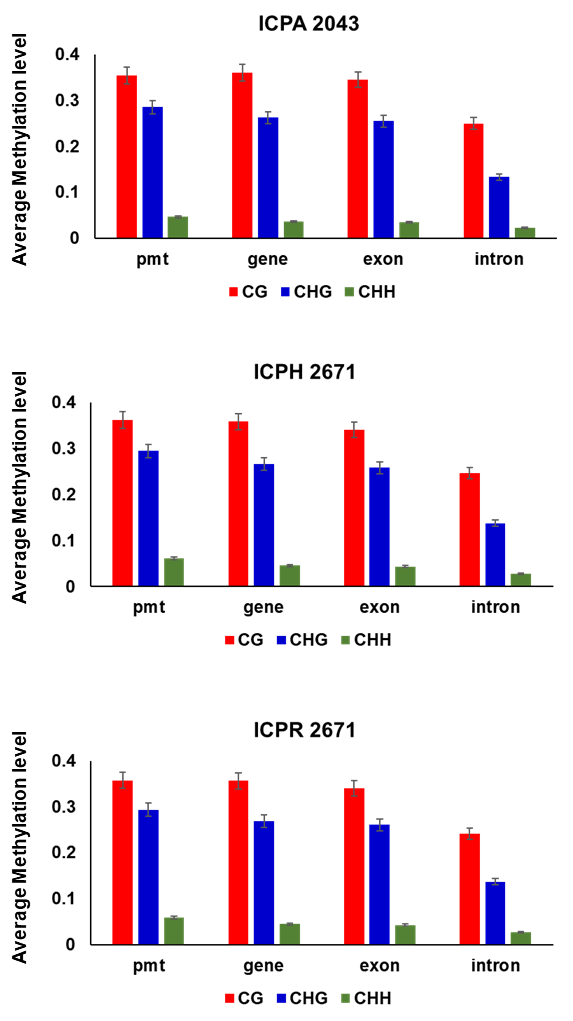


**Figure S3.** Canonical DNA methylation profiles of ICPH 2671 and its parental lines. The canonical gene structure is defined by four different features (promoter (pmt), genic (gene), exon and intron) denoted by the X-axis. The Y-axis represents the average methylation levels.


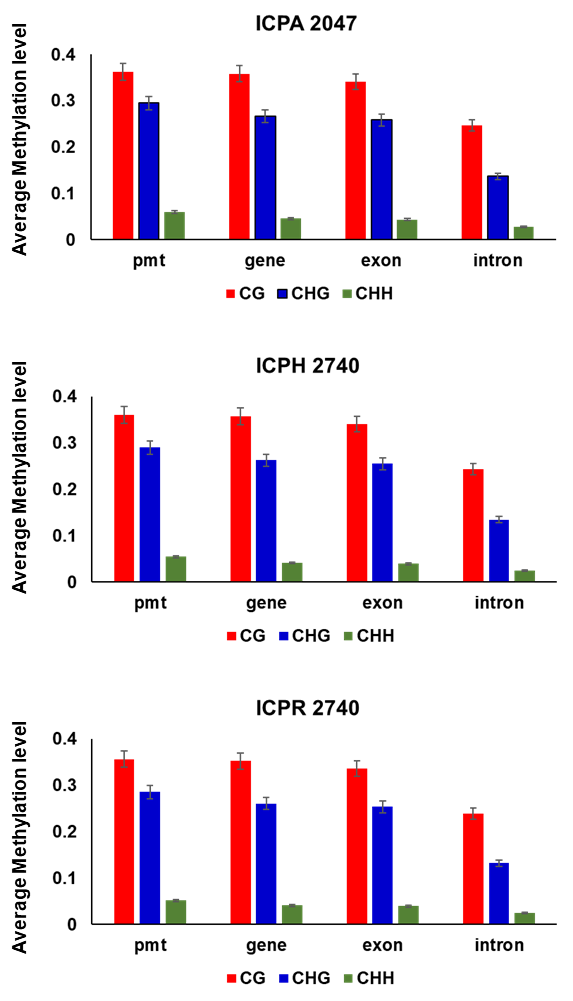


**Figure S4.** Canonical DNA methylation profiles of ICPH 2740 and its parental lines. The canonical gene structure is defined by four different features (promoter (pmt), genic (gene), exon and intron) denoted by the X-axis. The Y-axis represents the average methylation levels.


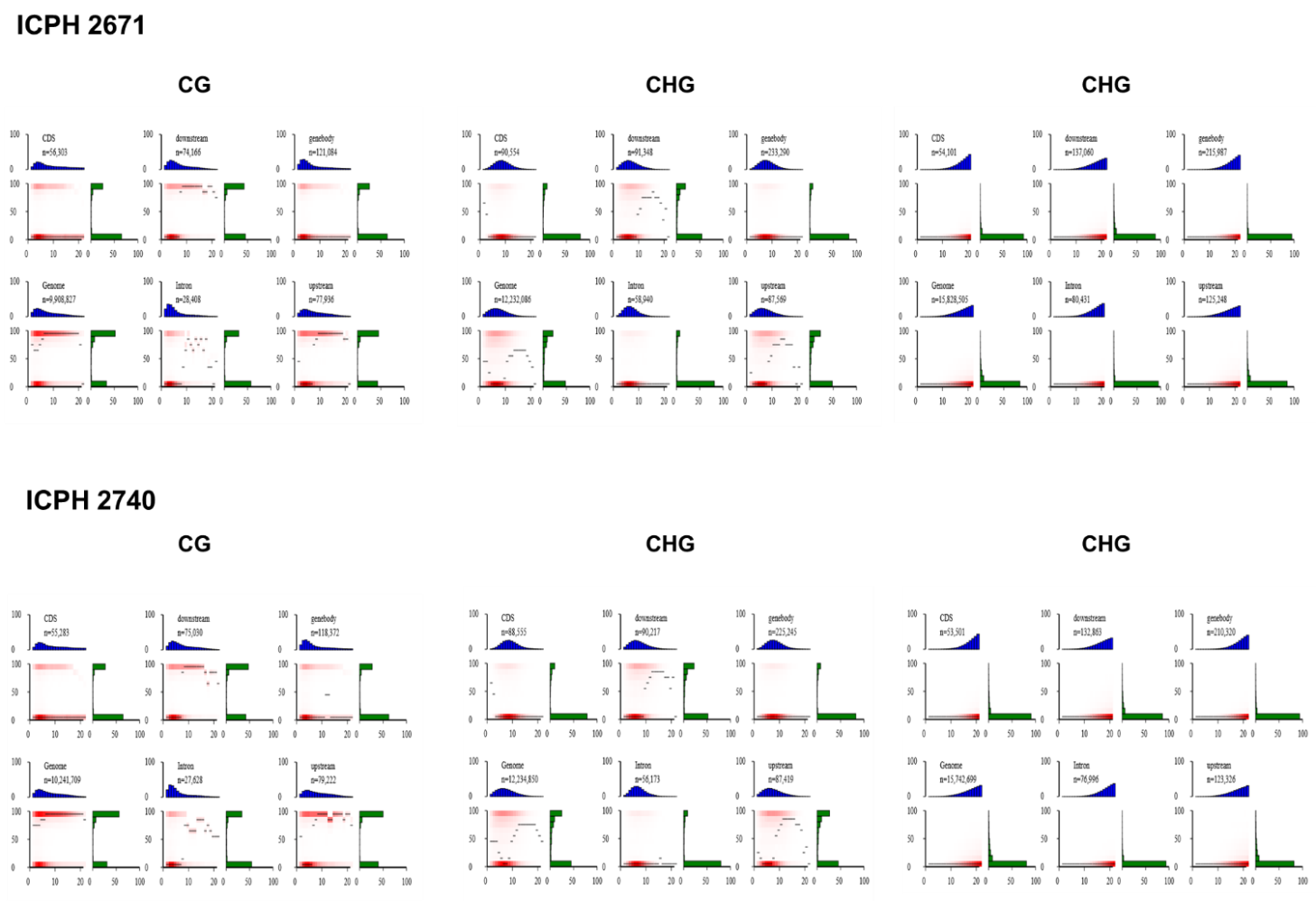


**Figure S5.** Heat maps show distinct methylation and CpGs (CG, CHG, CHH) density patterns for both the hybrid combinations across genome and at different regulatory units.


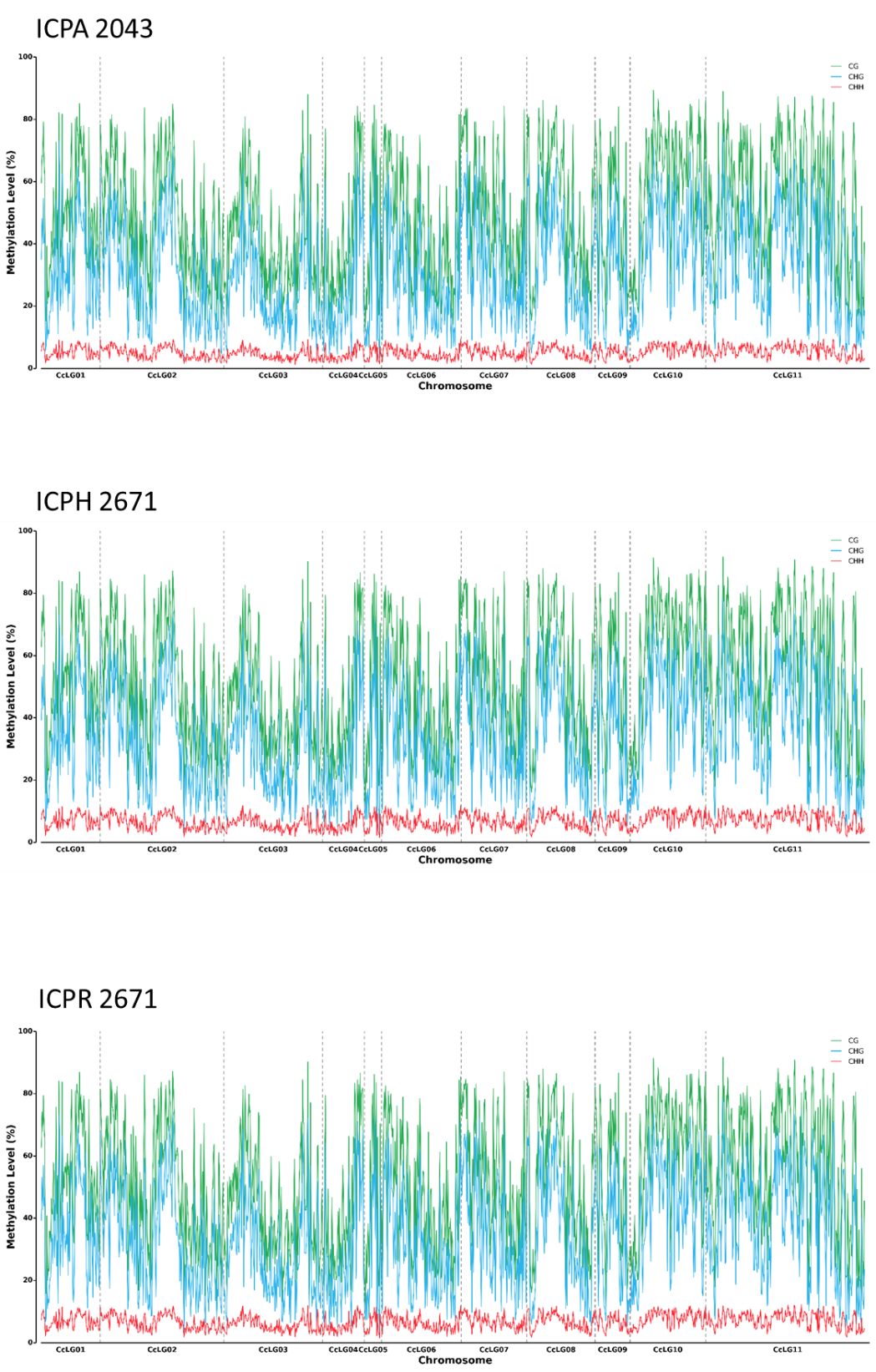


**Figure S6.** Methyl-cytosine density distribution of ICPH 2671 and its parental. The methyl-cytosine distribution density was showed as follows in different chromosomes (Green color represents CG, Blue color defines CHG and Red color defines CHH methylation density).


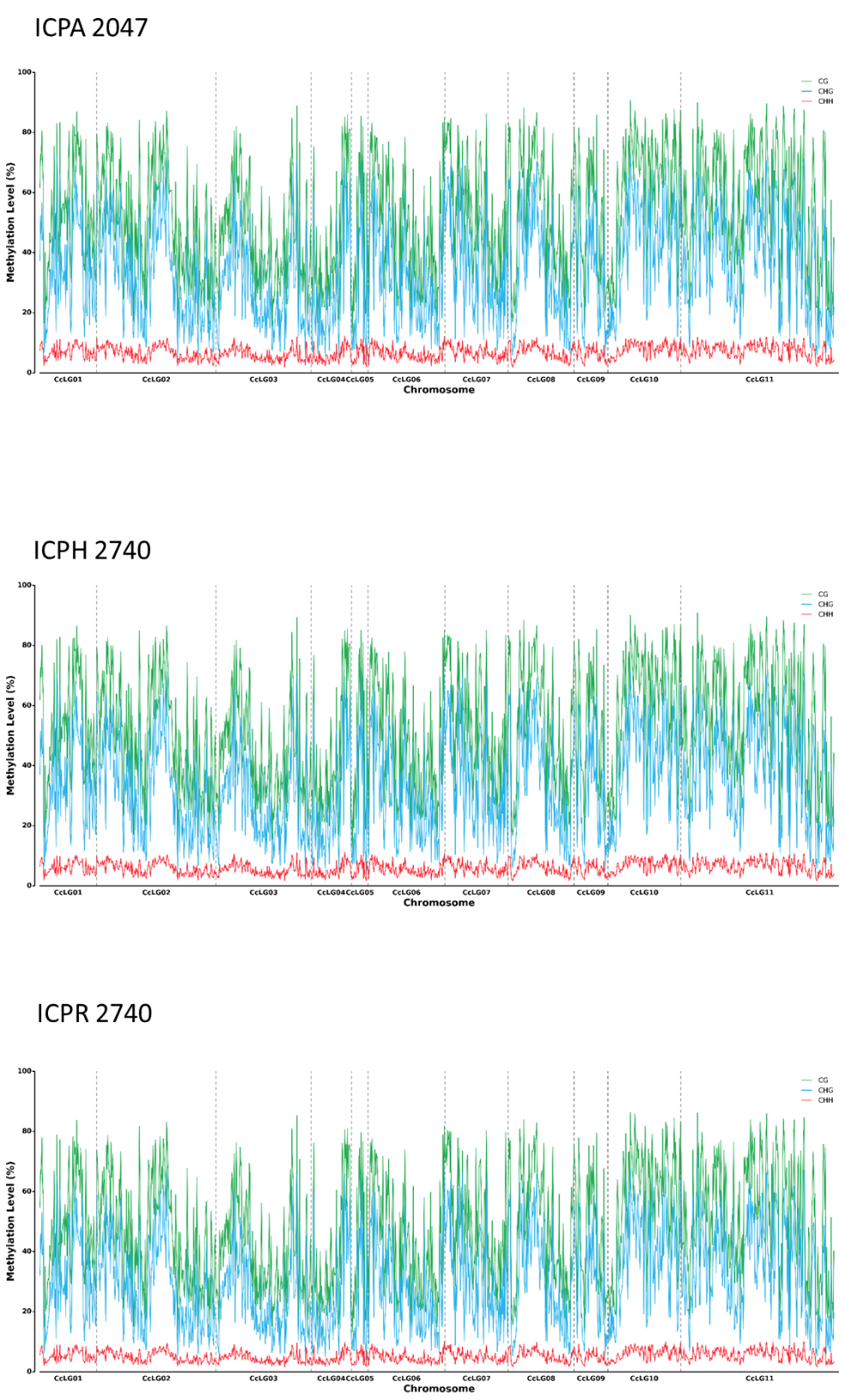


**Figure S7.** Methyl-cytosine density distribution of ICPH 2740 at different CG. The methyl-cytosine distribution density was showed as follows in different chromosomes. (Green color represents CG, Blue color defines CHG and Red color defines CHH methylation density).


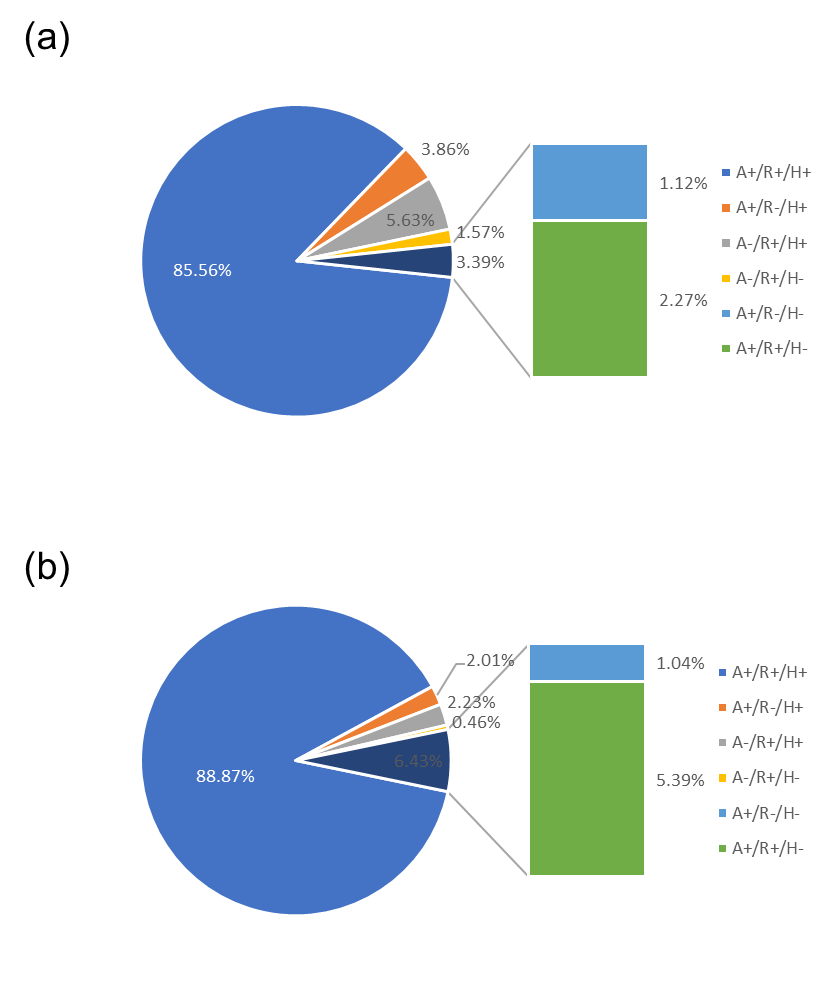


**Figure S8.** Comparative analysis of percent methylation contributed from parental lines in hybrid (a) ICPH 2671 and (b) ICPH 2740


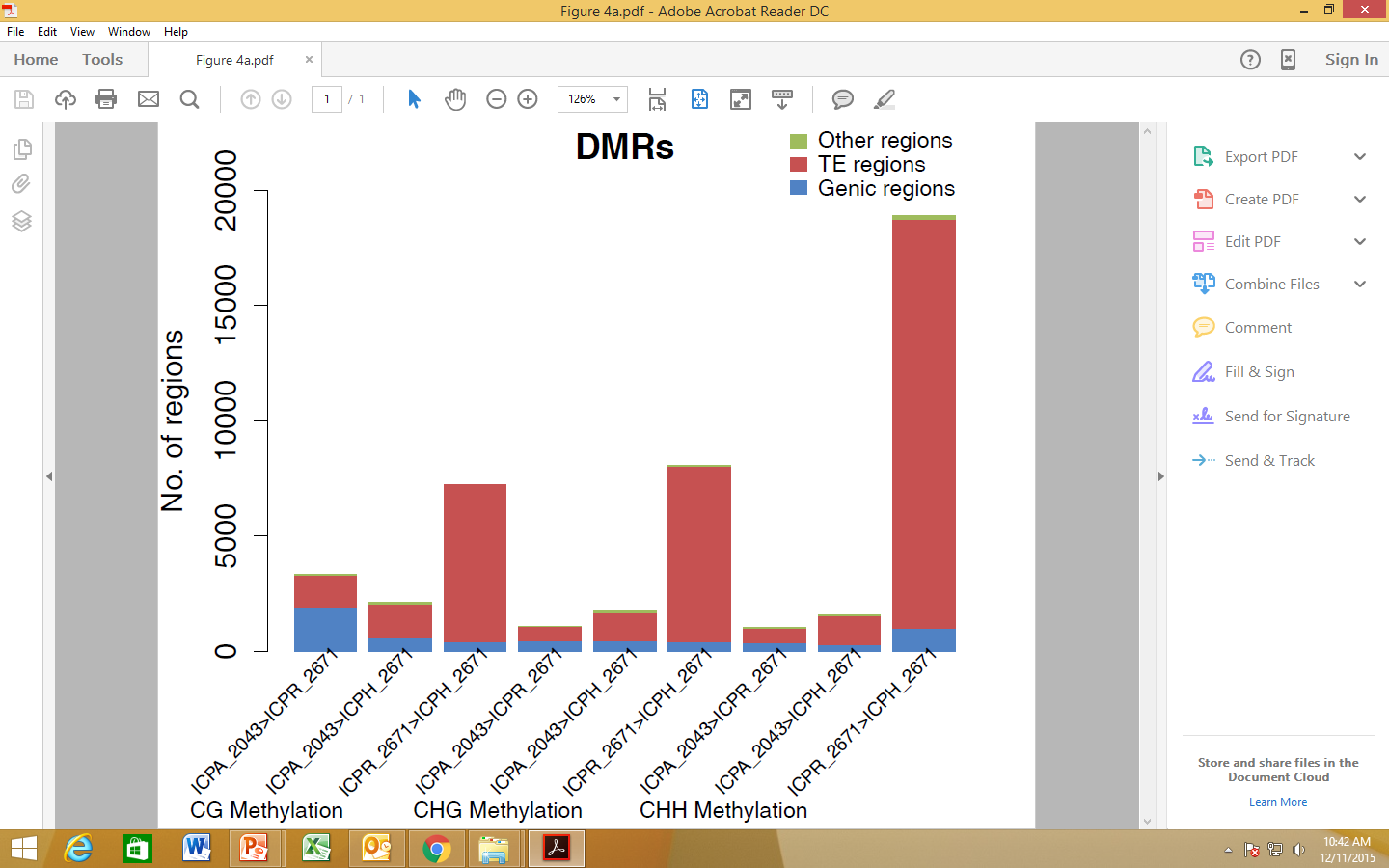


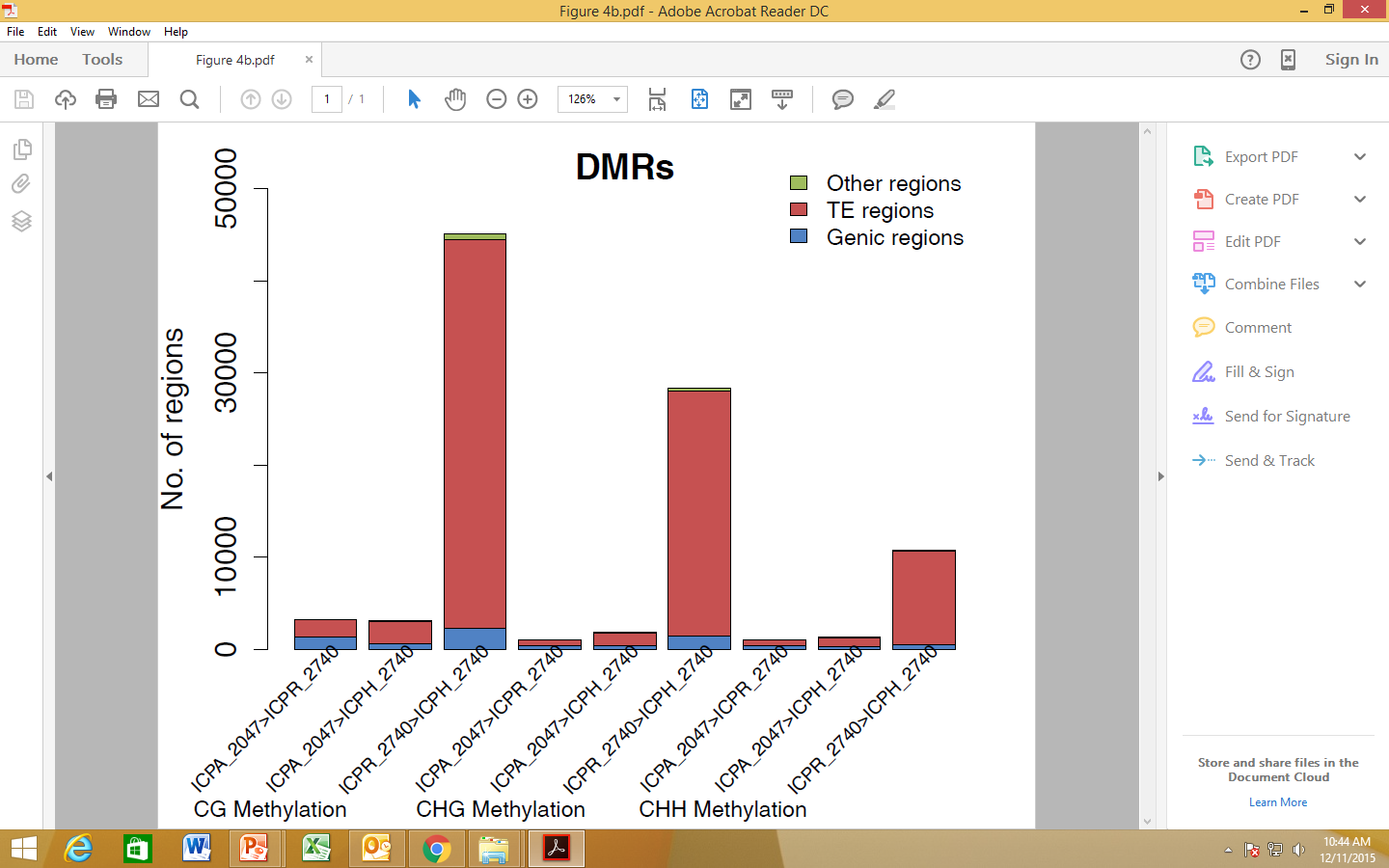


**Figure S9.** Distribution of DMRs of different cytosine contexts in different regions for (a) ICPH 2671 and (b) ICPH 2740 hybrid combinations.


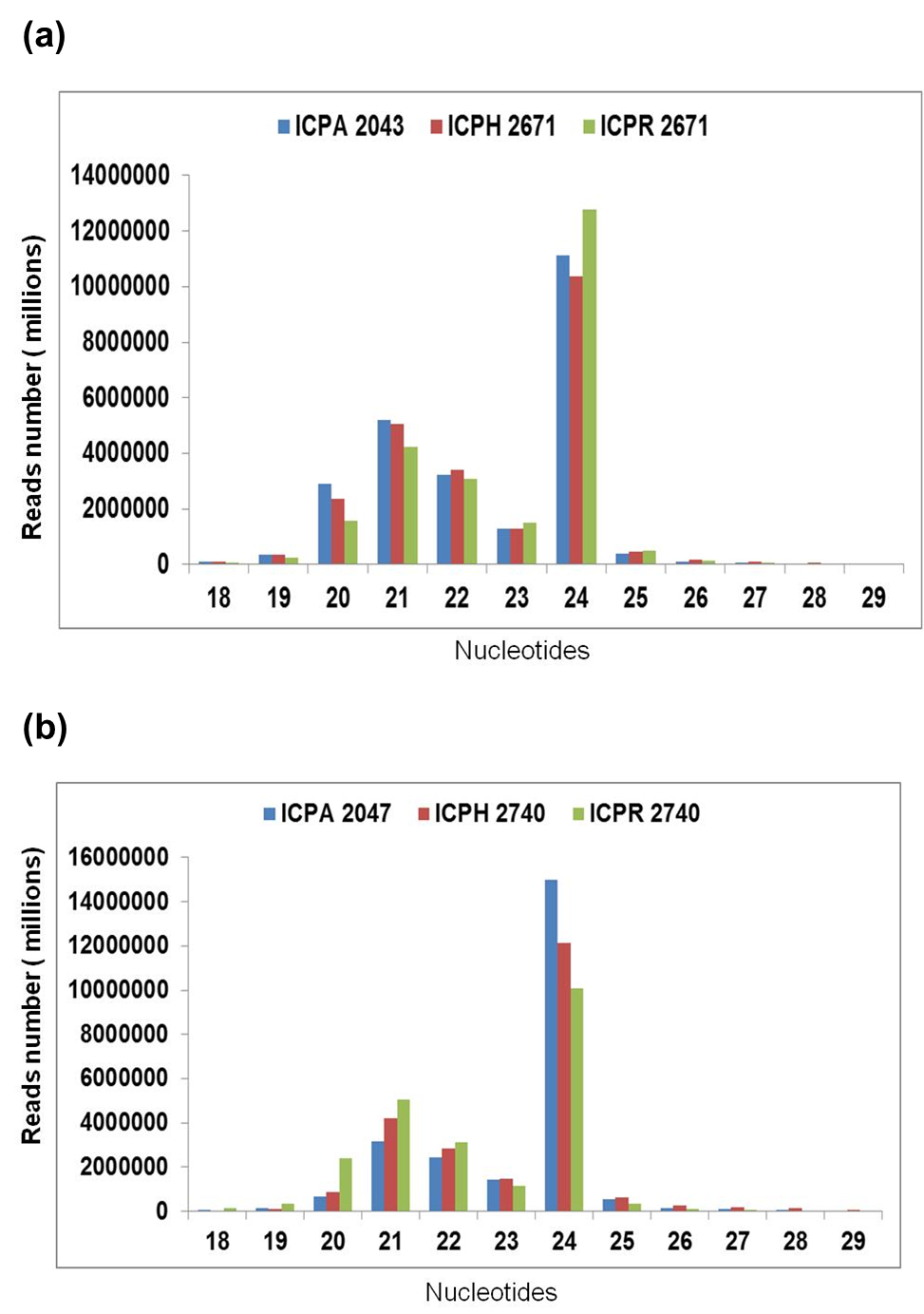


**Figure S10** Class distribution analysis of filtered reads in hybrids and parental lines showed that 21 and 24 nucleotide classes were the most abundant groups in (a) ICPH 2671 (b) and its parental lines.


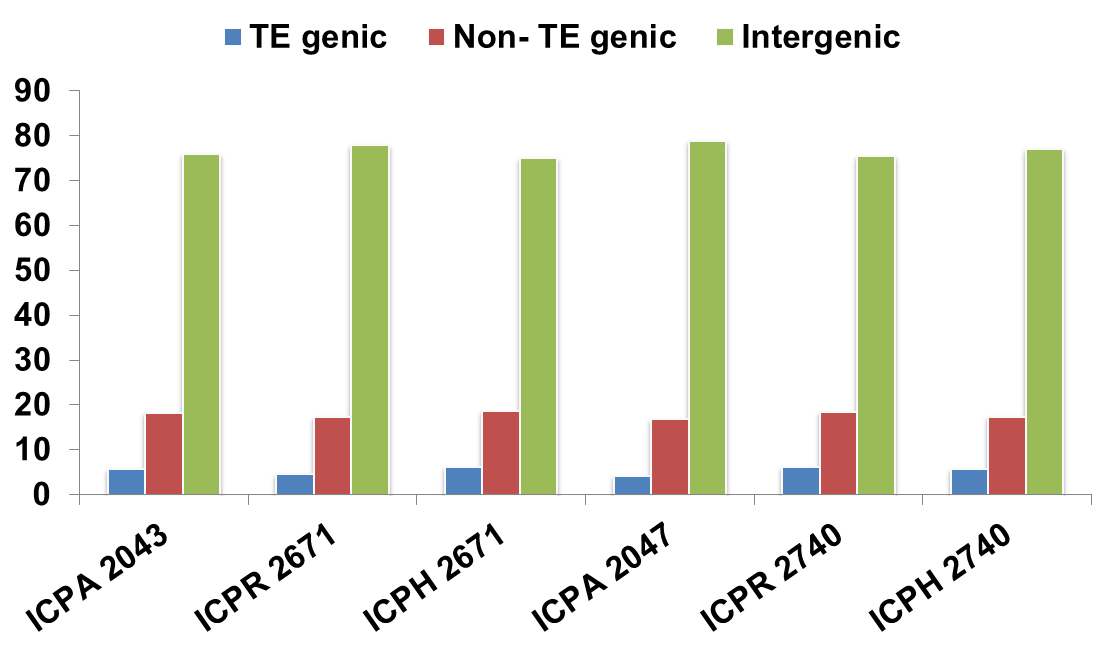


**Number of genes**

**Figure S11.** More enrichment of sRNA was observed in intergenic regions followed by non-TE related genes and TE-related genes in both the hybrids and their parental lines


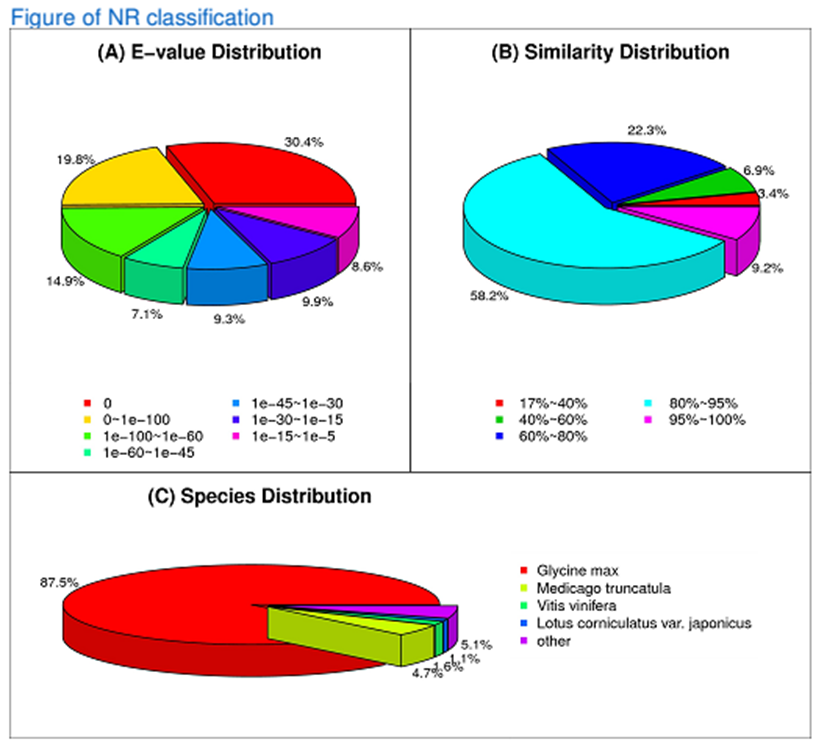


**Figure S12** NR Classification of Unigenes (A) The E- value distribution of the result of NR annotation. (B) The similarity distribution of the result of NR annotation. (C) The species distribution of the result of NR annotation


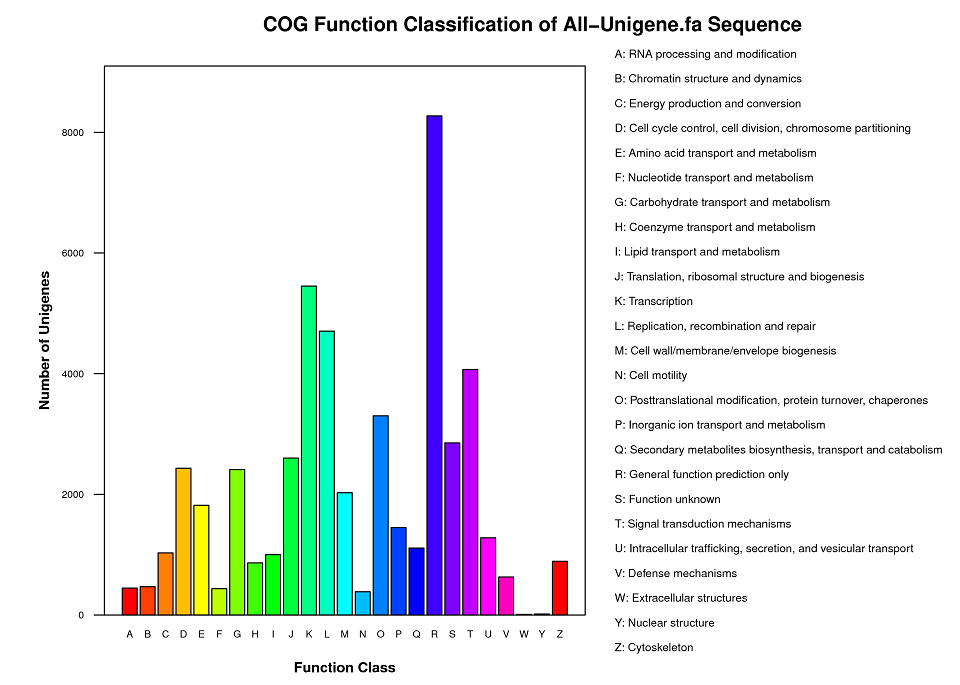


**Figure S13** COG function classification of Unigenes in All-Unigenes. The horizontal coordinates are function classes of COG, and the vertical coordinates are numbers of Unigenes in one class. The notation on the right is the full name of the functions in X-axis.


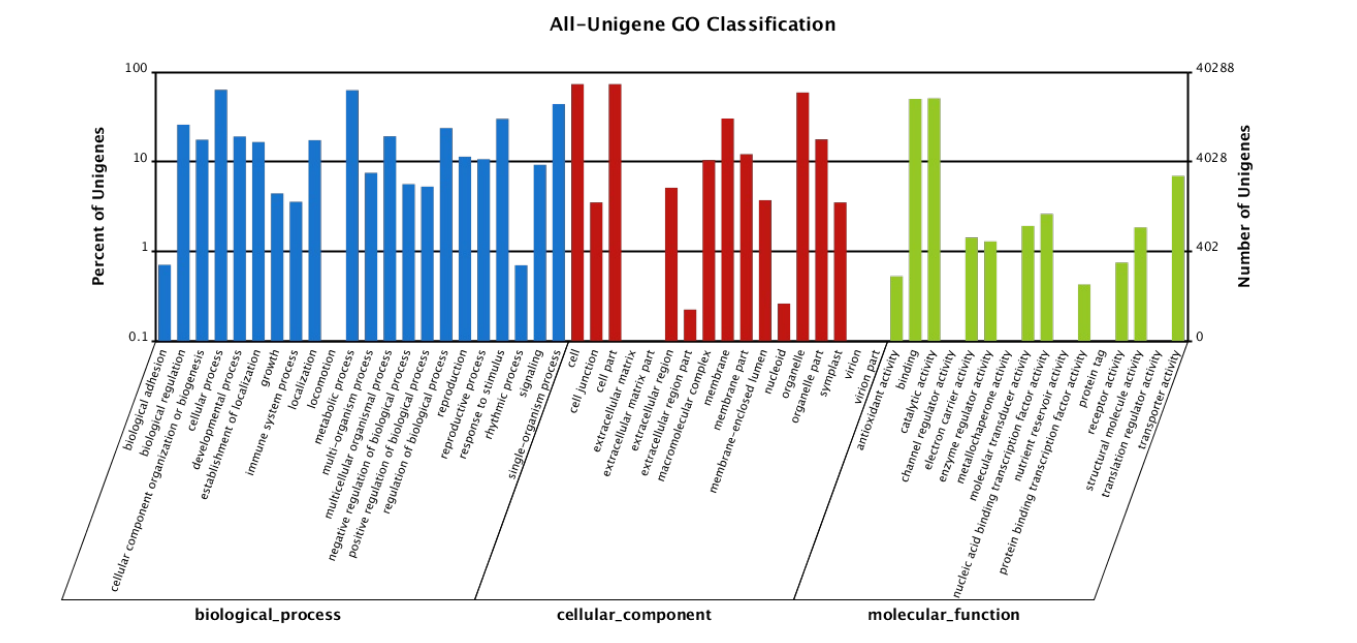


**Figure S14** GO classification analysis of Unigenes. GO functions are showed in X-axis. The right Y-axis shows the number of genes which have the GO function, and the left Y-axis shows the percentage


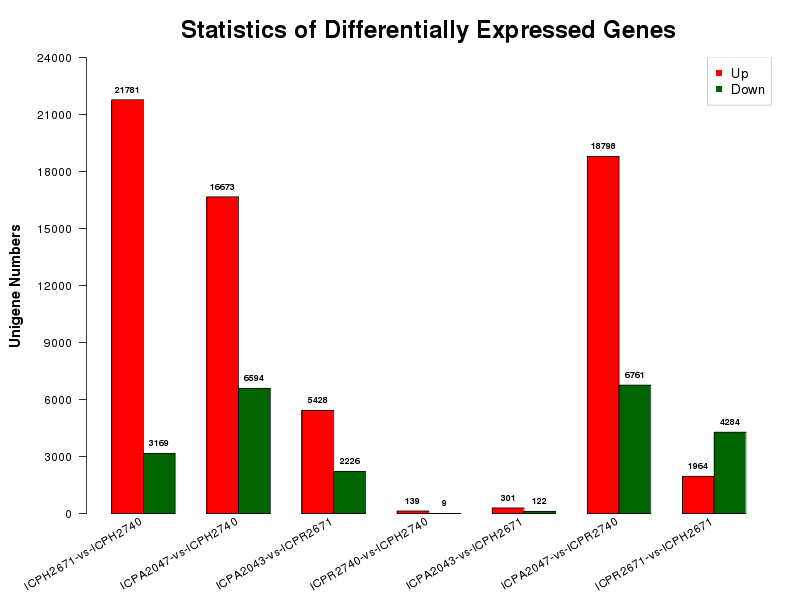


**Figure S15.** Number of DEGs identified between different combinations of hybrids and parental lines


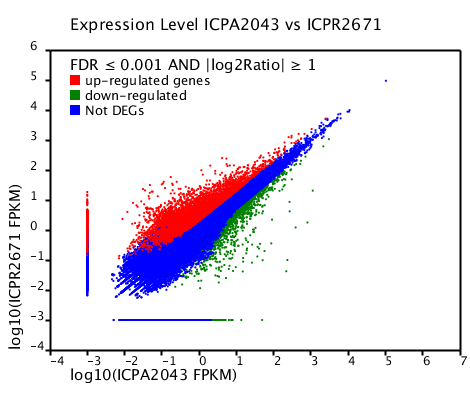


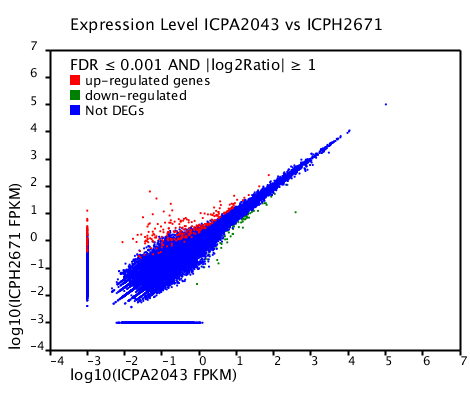


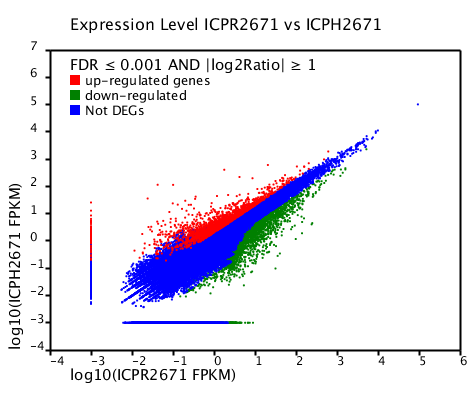


**Figure S16** Expression levels of genes (DEGs and non-DEGs) between different combinations of hybrid (ICPH 2671) and its parental lines (ICPA 2043 and ICPR 2671)


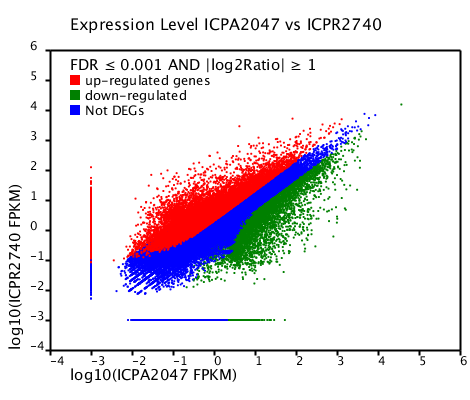


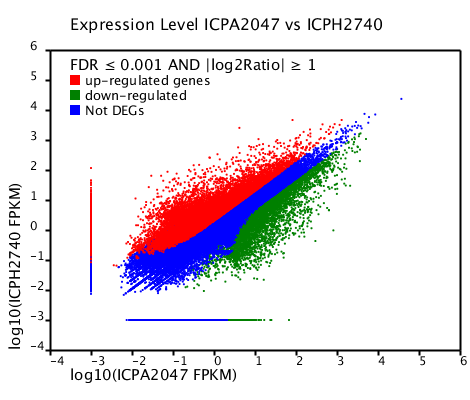


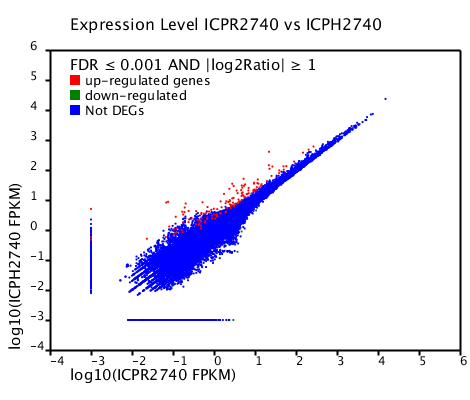


**Figure S17** Expression levels of genes (DEGs and non DEGs) between different combinations of hybrid (ICPH 2740) and its parental lines (ICPA 2047 and ICPR 2740)


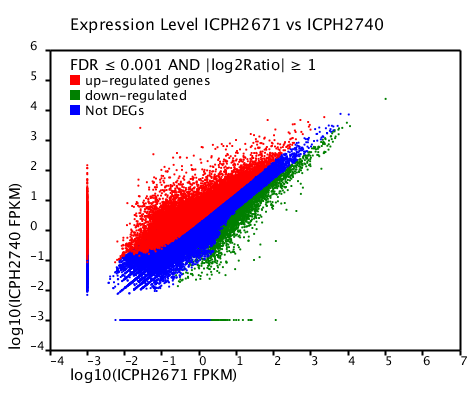


**Figure S18** Expression levels of genes (DEGs and non DEGs) between different hybrids (ICPH 2671 and ICPH 2740)


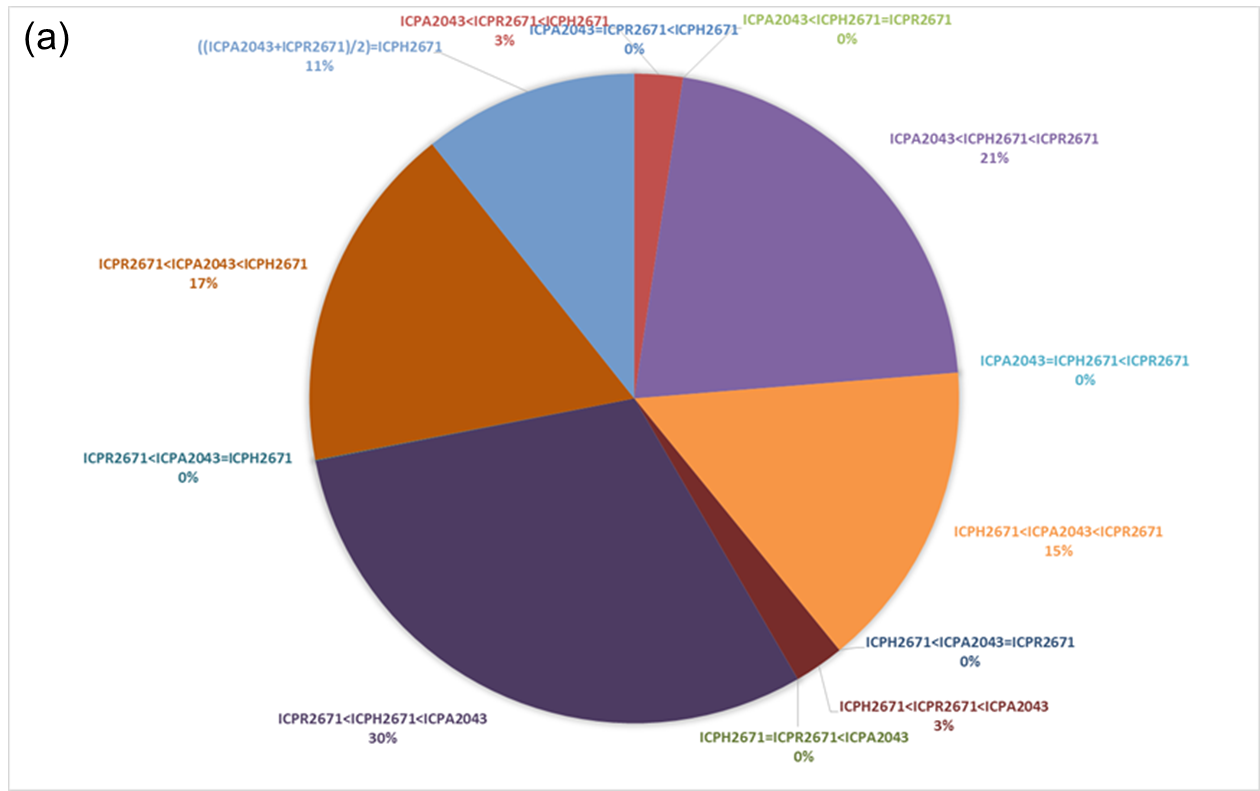


**
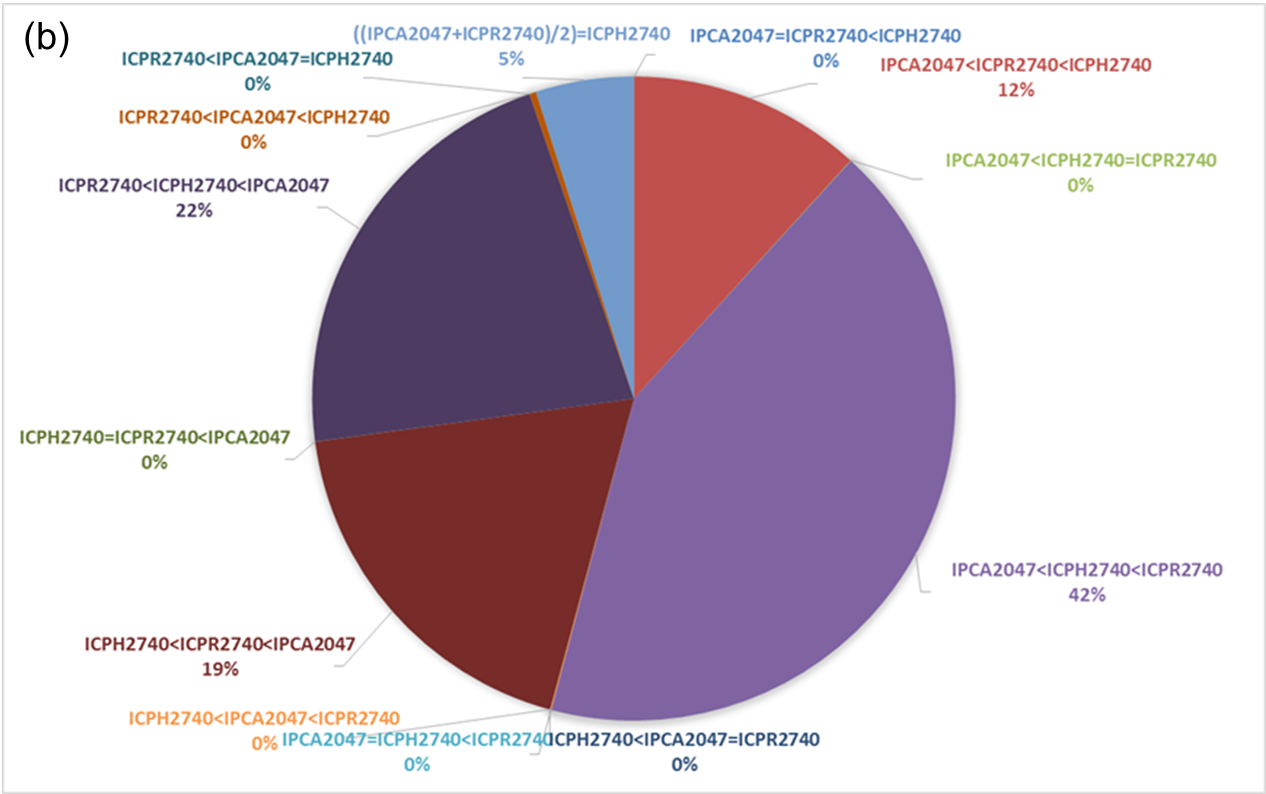
**

**Figure S19.** Classification of gene action in (a) ICPH 2671 and (b) ICPH 2740 hybrid combination based on the genome wide transcriptome data


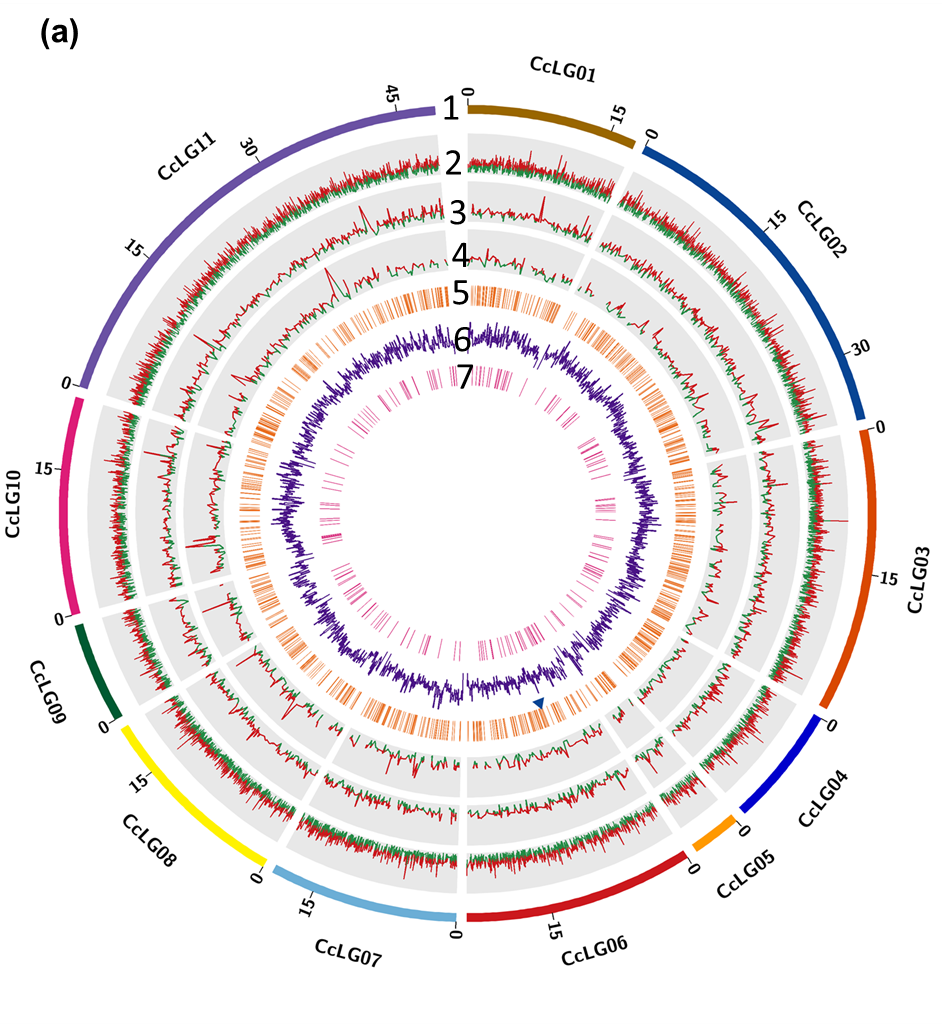


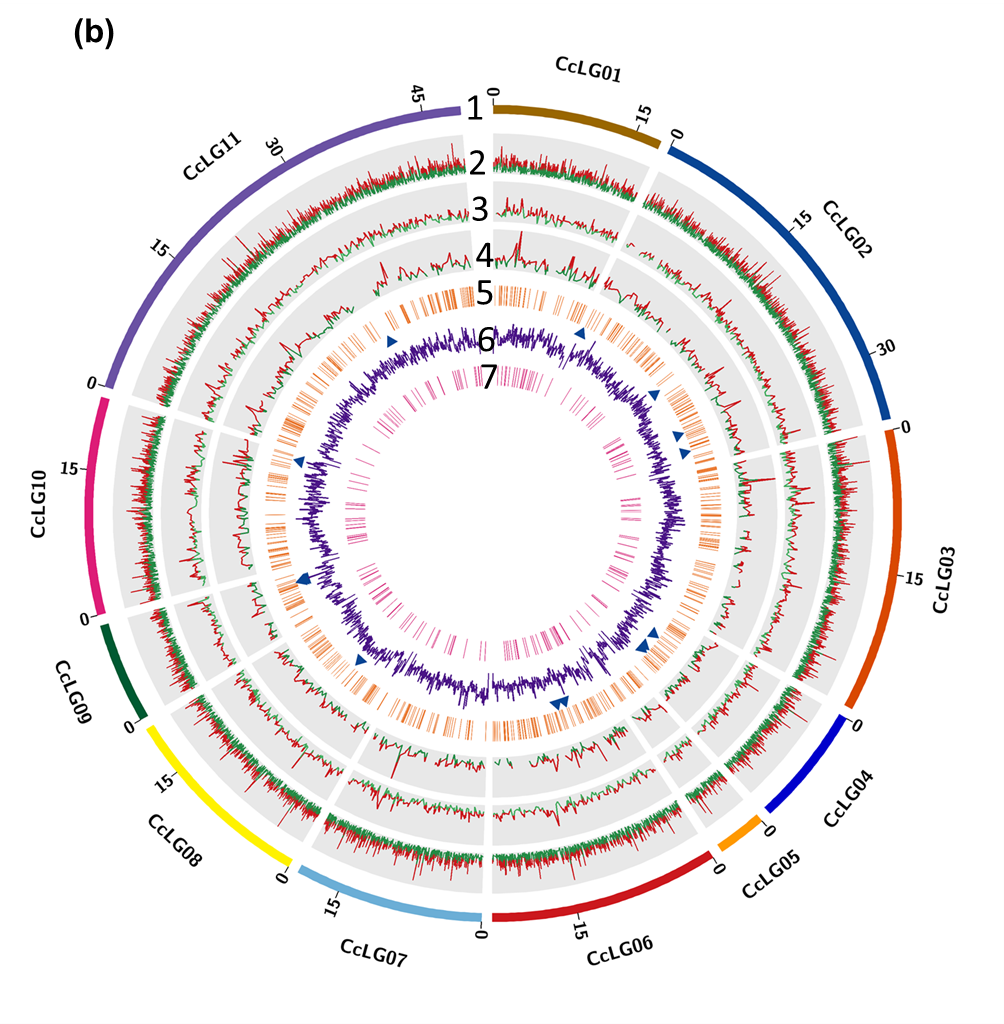
 **Figure S20.** Global cross-talk between genome wide epigenetic regulation, miRNA, sRNA and gene expression in realizing pigeonpea heterosis. (a) Cross talk between ICPH 2671 and (b) between ICPH 2740 combination. There are seven circles (label 1-7) in (a) and (b), the outermost circle (1) represents 11 pseudomolecules of *Cajanus cajan*, (2-4) Differentially methylated regions in CG, CHG and CHH context respectively, (5) Differentially expressed genes (DEGs), (6) 24nt sRNA frequency and (7) miRNAs location on genome. (c-d) Overview of the genetic regulatory network controlling flowering time with identified DMRs associated DEGs genes.

***Supplementary tables***

**Table S1.** Heterotic phenotypes of 15 and 30 days-old pigeonpea F_1_ hybrids relative to their parents

|  | 15 days after sowing | | | | | | 30 days-after sowing | | | | | |
| --- | --- | --- | --- | --- | --- | --- | --- | --- | --- | --- | --- | --- |
| Hybrids/parental lines | PW (g) | MPH (%) | SL (cm) | MPH  (%) | RL (cm) | MPH  (%) | PW (g) | MPH  (%) | SL (cm) | MPH  (%) | RL (cm) | MPH  (%) |
| *ICPH 2671 and its parental lines* | | | | | | | | | | | | |
| ICPA 2043 | 0.85±0.03 |  | 10.28±0.07 |  | 10.38±0.13 |  | 1.29±0.06 |  | 15.75±0.25 |  | 12.75±0.25 |  |
| ICPH 2671 | 0.98±0.04 | 19.51 | 12.26±0.20 | 21.56 | 11.17±0.21 | 16.53 | 1.41±0.05 | 22.07 | 18.50±0.50 | 21.31 | 13.68±0.12 | 16.42 |
| ICPR 2671 | 0.79±0.04 |  | 9.89±0.14 |  | 8.79±0.12 |  | 1.02±0.02 |  | 14.75±0.25 |  | 10.75±0.25 |  |
| *ICPH 2740 and its parental lines* | | | | | | | | | | | | |
| ICPA 2047 | 0.78±0.05 |  | 11.29±0.08 |  | 10.14±0.18 |  | 1.33±0.03 |  | 18.75±0.25 |  | 14.50±0.50 |  |
| ICPH 2740 | 0.90±0.02 | 21.62 | 11.68±0.09 | 16.85 | 10.63±0.14 | 18.50 | 1.51±0.08 | 26.89 | 19.85±0.50 | 20.30 | 15.25±0.25 | 17.37 |
| ICPR 2740 | 0.70±0.07 |  | 8.70±0.27 |  | 7.80 ±0.19 |  | 1.05±0.02 |  | 14.25±0.25 |  | 11.50±0.50 |  |

PW: Plant weight (g); SL: Shoot length (cm); Root length (cm); MPH: mid parent heterosis (%) ± (Standard error calculated from replicated data); ICPA (CMS or female parent); ICPH (Hybrid or F1) and ICPR (restorer or male parent)

**Table S2.** DNA methylation reads generated and alignment of parental lines and hybrids

| Samples | Raw reads  (millions) | Raw data  (Gb) | Reads mapped (millions) | Mapped data  (Gb) | Average map rate (%) | Whole genome average coverage depth (X) |
| --- | --- | --- | --- | --- | --- | --- |
| *ICPH 2671 and its parental lines* | | | | | | |
| ICPA 2043 | 186.1 | 16.8 | 170.1 | 15.3 | 91.4 | 25.3 |
| ICPH 2671 | 179.0 | 16.1 | 164.0 | 14.8 | 91.6 | 24.4 |
| ICPR 2671 | 178.1 | 16.0 | 165.1 | 14.9 | 92.7 | 24.5 |
| *ICPH 2740 and its parental lines* | | | | | | |
| ICPA 2047 | 185.0 | 16.6 | 169.8 | 15.3 | 91.8 | 25.2 |
| ICPH 2740 | 183.0 | 16.5 | 168.5 | 15.2 | 92.1 | 25.0 |
| ICPR 2740 | 221.9 | 20.0 | 205.2 | 18.5 | 92.5 | 30.5 |

**Table S3.** Pairwise comparison of DMRs among parents and their hybrids

| Methylation level | CG | | CHG | | CHH | |  |
| --- | --- | --- | --- | --- | --- | --- | --- |
|  | total | ratio | total | ratio | total | ratio |  |
| *ICPH 2671 and its parental lines* | | | | | | |  |
| ICPA 2043<ICPH 2671 | 1696 | 60.66% | 1916 | 52.22% | 44436 | 84.62% |  |
| ICPA 2043>ICPH 2671 | 1100 | 39.34% | 1753 | 47.78% | 8077 | 15.38% |  |
| ICPA 2043<ICPR 2671 | 2167 | 39.43% | 2355 | 52.56% | 37403 | 83.66% |  |
| ICPA 2043>ICPR 2671 | 3329 | 60.57% | 2126 | 47.44% | 7305 | 16.34% |  |
| ICPR 2671<ICPH 2671 | 2816 | 73.10% | 1980 | 55.80% | 20342 | 51.84% |  |
| ICPR 2671>ICPH 2671 | 1036 | 26.90% | 1569 | 44.20% | 18900 | 48.16% |  |
| *ICPH 2740 and its parental lines* | | | | | | |  |
| ICPA 2047<ICPH 2740 | 1149 | 53.15% | 1083 | 36.64% | 13893 | 32.87% |  |
| ICPA 2047>ICPH 2740 | 1013 | 46.85% | 1873 | 63.36% | 28374 | 67.13% |  |
| ICPA 2047<ICPR 2740 | 1846 | 36.32% | 3071 | 66.46% | 7897 | 14.92% |  |
| ICPA 2047>ICPR 2740 | 3237 | 63.68% | 1550 | 33.54% | 45040 | 85.08% |  |
| ICPR 2740<ICPH 2740 | 2263 | 67.82% | 2029 | 60.55% | 29272 | 73.15% |  |
| ICPR 2740>ICPH 2740 | 1074 | 32.18% | 1322 | 39.45% | 10747 | 26.85% |  |

Note: DMR stands for the Differentially methylated region and ICPA 2043 < ICPH 2671: DMRs in which ICPA 2043 is less methylated than ICPH 2671;

Similarly, ICPA 2043 > ICPH 2671: DMRs in which ICPA 2043 is more methylated than ICPH 2671

**Table S4.** List of common DMRs among parental lines and hybrid (*Datasets*)

**Table S5.** List of DMRs among parental lines and hybrid within the gene elements

| Hybrid combinations | Total DMRs | Total genic DMR | Percent genic DMR | Total gene associated DMR | | | | | |
| --- | --- | --- | --- | --- | --- | --- | --- | --- | --- |
|  |  |  |  | Gene associated DMRs | Percent gene associated DMRs | 2Kb upstream associated DMRs | Percent 2 kb upstream associated DMRs | 2 kb downstream associated DMRs | Percent 2 kb downstream associated DMRs |
| *ICPH 2671 and its parental lines* | | | | | | | | | |
| ICPA 2043/ICPR 2671 | 42300 | 12576 | 29.73 | 1233 | 9.80 | 5775 | 45.92 | 5568 | 44.27 |
| ICPA 2043/ICPH 2671 | 46347 | 11027 | 23.79 | 852 | 7.73 | 5169 | 46.88 | 5006 | 45.40 |
| ICPR 2671/ICPH 2671 | 35729 | 9815 | 27.47 | 921 | 9.38 | 4516 | 46.01 | 4378 | 44.61 |
| *ICPH 2740 and its parental lines* | | | | | | | | | |
| ICPA 2047/ICPR 2740 | 52065 | 12492 | 23.99 | 1042 | 8.34 | 5764 | 46.14 | 5686 | 45.52 |
| ICPA 2047/ICPH 2740 | 36417 | 9138 | 25.09 | 682 | 7.46 | 4276 | 46.79 | 4180 | 45.74 |
| ICPR 2740/ICPH 2740 | 38234 | 9427 | 24.66 | 782 | 8.30 | 4364 | 46.29 | 4281 | 45.41 |

**Table S6.** Classification of DMRs among parental lines and hybrids

| Genotypes | Methylation levels in parental lines | DMRs (13987) | |
| --- | --- | --- | --- |
|  |  | Interactive DMRs (13512) | Non-interactive DMRs (475) |
| *ICPH 2671 combinations* | | | |
| Trans-chromosomal methylation (TCM) | | | |
| ICPA 2043 | 2070 | 7884 | 297 |
| ICPR 2671 | 5774 |  |  |
| ICPH 2671 |  |  |  |
| Trans-chromosomal demethylation (TCdM) | | | |
| ICPA 2043 | 1335 | 5668 | 178 |
| ICPR 2671 | 4333 |  |  |
| ICPH 2671 |  |  |  |
|  |  | DMRs (15132) | |
| *ICPH 2740 combinations* |  | Interactive DMRs (14737) | Non-interactive DMRs (395) |
| Trans-chromosomal methylation (TCM) | | | |
| ICPA 2047 | 5121 | 6269 | 243 |
| ICPR 2740 | 1391 |  |  |
| ICPH 2740 |  |  |  |
| Trans-chromosomal demethylation (TCdM) | | | |
| ICPA 2047 | 7391 | 8468 | 152 |
| ICPR 2740 | 1229 |  |  |
| ICPH 2740 |  |  |  |

**Table S7.** Statistics of small RNA sequencing data production

| Genotype | Raw reads  (millions) | Clean reads  (millions) | Clean reads (%) | Reads mapped to the genome (million) | sRNA reads^†^ (millions) | Unique sRNA reads (millions) |
| --- | --- | --- | --- | --- | --- | --- |
| *ICPH 2671 and its parental lines* | | | | | | |
| ICPA 2043 | 24.95 | 24.76 | 99.60 | 20.83 | 13.79 | 5.51 |
| ICPH 2671 | 23.91 | 23.74 | 99.63 | 20.01 | 12.44 | 5.16 |
| ICPR 2671 | 24.25 | 24.09 | 99.69 | 20.55 | 13.28 | 5.60 |
| *ICPH 2740 and its parental lines* | | | | | | |
| ICPA 2047 | 23.91 | 23.73 | 99.61 | 19.87 | 13.97 | 6.08 |
| ICPH 2740 | 23.05 | 22.91 | 99.75 | 19.48 | 12.19 | 5.28 |
| ICPR 2740 | 23.00 | 22.79 | 99.41 | 19.46 | 12.33 | 4.99 |

**^†^**sRNA reads obtained after removing reads mapped to rRNA, tRNA, small nuclear RNA and nucleolar RNA

**Table S8**. Statistics of small RNA distribution identified in genic and flanking (up and downstream)

| Genotype | Total | Gene | Cut off (18) | | Cut off (10) | |
| --- | --- | --- | --- | --- | --- | --- |
|  |  |  | Upstream  (2 Kb) | Downstream  (2 Kb) | Upstream  (2 Kb) | Downstream  (2 Kb) |
| *ICPH 2671 and its parental lines* | | | | | | |
| ICPA 2043 | 21297167 | 1277537 | 237221 | 1190996 | 239500 | 1214498 |
| ICPH 2671 | 20003690 | 1204904 | 222111 | 1118404 | 224047 | 1139979 |
| ICPR 2671 | 21332887 | 1261482 | 239532 | 1179772 | 241197 | 1199400 |
| *ICPH 2740 and its parental lines* | | | | | | |
| ICPA 2047 | 23037182 | 1333827 | 259293 | 1263419 | 261340 | 1285395 |
| ICPH 2740 | 20092163 | 1206670 | 228869 | 1130299 | 230291 | 1147209 |
| ICPR 2740 | 20313121 | 1200510 | 225634 | 1113509 | 228092 | 1137402 |

**Table S9.** Association of DMRs and sRNA among ICPH 2671 and its parental lines

| Genotypes | Methylation levels in parental lines | Comparison between parental sRNA with DMRs (13987) | | | | sRNA and DNA methylation interaction in different cytosine context | | | | | | Common sRNA present in methylated regions | Percent of parents contributed equally towards hybrid sRNA | Unique sRNA | Positive positions^Ɨ^ | Negative positions^‡^ |
| --- | --- | --- | --- | --- | --- | --- | --- | --- | --- | --- | --- | --- | --- | --- | --- | --- |
|  |  |  |  |  |  | CG | | CHG | | CHH | |  |  |  |  |  |
|  |  | Interactive DMRs  (13512) | | Non-interactive DMRs (475) | |  |  |  |  |  |  |  |  |  |  |  |
|  |  | sRNA present in any of the parents | sRNA present in ICPH 2671 | sRNA present in any of the parents | sRNA present in ICPH 2671 | -sRNA | +sRNA | -sRNA | +sRNA | -sRNA | +sRNA |  |  |  |  |  |
| Trans-chromosomal methylation (TCM) (I: 7844 and NI: 297) | | | | | | | | | | | | | | | | |
| ICPA 2043 | 2070 | 3372  (42.98%) | 2722 (81.81%) | 117  (39.39%) | 92  (78.63%) | 905 | 516 | 516 | 332 | 3579 | 2006 | 2353 |  | 332 | 128 | 159  (5.84%) |
| ICPR 2671 | 5774 |  |  |  |  | 912 | 509 | 492 | 346 | 3542 | 2043 |  |  | 386 | 144 |  |
| ICPH 2671 |  |  |  |  |  | 928 | 493 | 524 | 314 | 3670 | 1906 |  | 86.44 % | 97 |  |  |
| Trans-chromosomal demethylation (TCdM) (I: 5668 and NI: 178) | | | | | | | | | | | | | | | | |
| ICPA 2043 | 1335 | 2505  (44.19%) | 2092  (83.51%) | 86  (48.31%) | 72  (83.72%) | 468 | 278 | 343 | 229 | 2708 | 1642 | 1782 |  | 250 | 124 | 117  (5.59%) |
| ICPR 2671 | 4333 |  |  |  |  | 457 | 245 | 342 | 201 | 2683 | 1453 |  |  | 284 | 117 |  |
| ICPH 2671 |  |  |  |  |  | 474 | 272 | 349 | 223 | 2753 | 1597 |  | 85.18 % | 69 |  |  |

**Table S10.** Association of DMRs and sRNA among ICPH 2740 and its parental lines

| Genotypes | Methylation levels in parental lines | Comparison between parental sRNA with DMRs (15132) | | | | sRNA and DNA methylation interaction in different cytosine context | | | | | | Common sRNA present in methylated regions | Percent of parents contributed equally towards hybrid sRNA | Unique sRNA | Positive positions^Ɨ^ | Negative positions^‡^ |
| --- | --- | --- | --- | --- | --- | --- | --- | --- | --- | --- | --- | --- | --- | --- | --- | --- |
|  |  |  |  |  |  | CG | | CHG | | CHH | |  |  |  |  |  |
|  |  | Interactive DMRs  (14737) | | Non-interactive DMRs (395) | |  | |  | |  | |  |  |  |  |  |
|  |  | sRNA present in any of the parents | sRNA present in ICPH 2740 | sRNA present in any of the parents | sRNA present in ICPH 2740 | -sRNA | +sRNA | -sRNA | +sRNA | -sRNA | +sRNA |  |  |  |  |  |
| Trans-chromosomal methylation (TCM) (I: 6269 and NI: 243) | | | | | | | | | | | | | | | | |
| ICPA 2047 | 5121 | 2782  (33.84%) | 2162 (77.71%) | 96  (39.50%) | 79  (82.22%) | 721 | 461 | 490 | 282 | 2718 | 1597 | 1806 | 83.53% | 457 | 206 | 77 (3.56%) |
| ICPR 2740 | 1391 |  |  |  |  | 773 | 409 | 520 | 252 | 2893 | 1422 |  |  | 200 | 121 |  |
| ICPH 2740 |  |  |  |  |  | 734 | 448 | 499 | 273 | 2761 | 1554 |  |  | 142 |  |  |
| Trans-chromosomal demethylation (TCdM) (I: 8468 and NI: 152) | | | | | | | | | | | | | | | | |
| ICPA 2047 | 7391 | 3793  (44.79%) | 2967 (78.22%) | 74  (48.68%) | 60  (81.08%) | 480 | 280 | 323 | 221 | 4407 | 2757 | 2541 | 85.64% | 605 | 250 | 112  (3.77%) |
| ICPR 2740 | 1229 |  |  |  |  | 508 | 252 | 353 | 191 | 4704 | 2460 |  |  | 250 | 120 |  |
| ICPH 2740 |  |  |  |  |  | 481 | 279 | 338 | 206 | 4530 | 2634 |  |  | 208 |  |  |

**Table S11.** Comparative analysis of small RNA (sRNA) and DNA methylation in the different CG context

| DMRs-small RNA | ICPH 2671 and its parental lines | | | ICPH 2740 and its parental lines | | |
| --- | --- | --- | --- | --- | --- | --- |
|  | ICPA 2043 | ICPH 2671 | ICPR 2671 | ICPA 2047 | ICPH 2740 | ICPR 2740 |
| CG_sRNA | 0.94 | 0.95 | 0.94 | 0.94 | 0.95 | 0.94 |
| CG_sRNA_noRNA | 0.59 | 0.62 | 0.62 | 0.63 | 0.62 | 0.56 |
| CG_total | 0.62 | 0.65 | 0.65 | 0.65 | 0.65 | 0.58 |
|  |  |  |  |  |  |  |
| CHG_sRNA | 0.77 | 0.78 | 0.79 | 0.78 | 0.78 | 0.77 |
| CHG_sRNA_noRNA | 0.39 | 0.43 | 0.43 | 0.43 | 0.42 | 0.37 |
| CHG_total | 0.42 | 0.46 | 0.46 | 0.46 | 0.45 | 0.39 |
|  |  |  |  |  |  |  |
| CHH_sRNA | 0.12 | 0.15 | 0.15 | 0.15 | 0.14 | 0.14 |
| CHH_sRNA_noRNA | 0.06 | 0.08 | 0.08 | 0.08 | 0.08 | 0.06 |
| CHH_total | 0.42 | 0.46 | 0.46 | 0.46 | 0.45 | 0.39 |

**Table S12.** List of differentially expressed miRNA and their target expression in ICPH 2671 and ICPH 2740 hybrid combinations (Dataset)

**Table S13**. Statistics of RNA sequencing data production

| Samples | Total raw reads (millions) | Total clean reads (millions) | Total clean nucleotides (bp) | Q20 percentage | N percentage | GC percentage |
| --- | --- | --- | --- | --- | --- | --- |
| *ICPH 2671 and its parental lines* | | | | | | |
| ICPA 2043 | 43.40 | 40.58 | 7,305,719,400 | 97.44% | 0.00% | 44.97% |
| ICPH 2671 | 43.58 | 40.72 | 7,330,869,900 | 97.38% | 0.00% | 46.54% |
| ICPR 2671 | 44.25 | 41.32 | 7,438,659,120 | 97.33% | 0.00% | 46.02% |
| *ICPH 2740 and its parental lines* | | | | | | |
| ICPA 2047 | 43.19 | 40.24 | 7,244,756,640 | 97.35% | 0.00% | 46.50% |
| ICPR 2740 | 41.21 | 38.45 | 6,922,480,140 | 97.43% | 0.00% | 45.80% |
| ICPH 2740 | 44.62 | 41.58 | 7,485,192,720 | 97.47% | 0.00% | 44.90% |

Total reads and Total nucleotides are actually clean reads and clean nucleotides; Total nucleotides should be more than contract provision percentage is the proportion of nucleotides with quality value larger than 20; N percentage is proportion of unknown nucleotides in clean reads; GC proportion of guanine and cytocin nucleotides among total nucleotides.

**Table S14.** Details of the development of contigs of parental lines and hybrids

| Samples | Total no contigs | Total length (bp) | Mean length (bp) | N50 |
| --- | --- | --- | --- | --- |
| *ICPH 2671 and its parental lines* | | | | |
| ICPA 2043 | 50,673 | 30,423,877 | 600 | 1271 |
| ICPH 2671 | 53,811 | 31,028,933 | 577 | 1238 |
| ICPR 2671 | 61,825 | 32,675,462 | 529 | 1143 |
| *ICPH 2740 and its parental lines* | | | | |
| ICPA 2047 | 61,012 | 35,096,545 | 575 | 1239 |
| ICPH 2740 | 78,587 | 39,670,381 | 505 | 1099 |
| ICPR 2740 | 80,122 | 40,521,651 | 506 | 1097 |

| Samples | Total no unigenes | Total length (bp) | Mean length (bp) | N50 | Total consensus sequences | Distinct clusters | Distinct singletons |
| --- | --- | --- | --- | --- | --- | --- | --- |
| *ICPH 2671 and its parental lines* | | | | | | | |
| ICPA 2043 | 32116 | 35012616 | 1090 | 1638 | 32116 | 10469 | 21647 |
| ICPH 2671 | 33506 | 36067504 | 1076 | 1634 | 33506 | 11020 | 22486 |
| ICPR 2671 | 38806 | 41606298 | 1072 | 1656 | 38806 | 13801 | 25005 |
| *ICPH 2740 and its parental lines* | | | | | | | |
| ICPA 2047 | 38133 | 41472855 | 1088 | 1639 | 38133 | 13086 | 25047 |
| ICPH 2740 | 52070 | 60548712 | 1163 | 1812 | 52070 | 21542 | 30528 |
| ICPR 2740 | 53776 | 62936350 | 1170 | 1811 | 53776 | 22643 | 31133 |
| Total | 63825 | 84662317 | 1326 | 1986 | 63825 | 29431 | 34394 |

**Table S15.** Details of the development of *de-novo* assemblies of parental lines and hybrids

**Table S16.** Details of annotations of unigenes against different databases

| Sequence file | NR | NT | Swiss-Prot | KEGG | COG | GO | ALL |
| --- | --- | --- | --- | --- | --- | --- | --- |
| All Unigene (63,825) | 49,775 | 52,941 | 34,151 | 31,644 | 22,230 | 40,288 | 53,996 |

Nr: non-redundant protein; NT: non-redundant nucleotide, Swiss-Prot protein database, KEGG: Kyoto Encyclopedia of Genes and Genomes Pathway database, COG: Clusters of Orthologous Groups; GO: database Gene Ontology

**Table S17.** List of differentially expressed genes (DEGs) between parental lines and hybrids

| Combinations of genotypes | Total DEGs | Up-regulations | Down-regulation |
| --- | --- | --- | --- |
| *ICPH 2671 and its parental lines* | | | |
| ICPA 2043/ICPR 2671 | 7185 | 5066 | 2119 |
| ICPA 2043/ICPH 2671 | 378 | 256 | 122 |
| ICPR 2671/ICPH 2671 | 6022 | 1884 | 4138 |
| *ICPH 2740 and its parental lines* | | | |
| ICPA 2047/ICPR 2740 | 23956 | 17646 | 6310 |
| ICPA 2047/ICPH 2740 | 15434 | 15001 | 433 |
| ICPR 2740/ICPH 2740 | 144 | 136 | 8 |
| *Between hybrids* | | | |
| ICPH 2671/ICPH 2740 | 24950 | 21781 | 3169 |

| **Table S18**. Statistical analysis and classification of genes based on gene action | | | |
| --- | --- | --- | --- |
| Class | Expression pattern | Total no. of genes | Gene action |
| Hybrid combination 1 (ICPH 2671) | | | |
| 1 | ICPA2043=ICPR2671<ICPH2671 | 0 | +ve over dominance |
| 2 | ICPA2043<ICPR2671<ICPH2671 | 174 (2.41 %) | +ve over dominance |
| 3 | ICPA2043<ICPR2671=ICPH2671 | 0 | Dominance |
| 4 | ICPA2043<ICPH2671<ICPR2671 | 1540 (21.32 %) | Partial dominance |
| 5 | ICPA2043=ICPH2671<ICPR2671 | 0 | -ve dominance |
| 6 | ICPH2671<ICPA2043<ICPR2671 | 1111 (15.38 %) | -ve over dominance |
| 7 | ICPH2671<ICPA2043=ICPR2671 | 0 | Dominance |
| 8 | ICPH2671<ICPR2671<ICPA2043 | 180 (2.49) % | -ve over dominance |
| 9 | ICPH2671=ICPR2671<ICPA2043 | 0 | -ve dominance |
| 10 | ICPR2671<ICPH2671<ICPA2043 | 2190 (30.32 %) | Partial dominance |
| 11 | ICPR2671<ICPA2043=ICPH2671 | 3 | Dominance |
| 12 | ICPR2671<ICPA2043<ICPH2671 | 1253 (17.32 %) | +ve over dominance |
| 13 | ((ICPA2043+ICPR2671)/2) =ICPH2671 | 772 (10.69%) | Additive |
| Total | | 7223 |  |
| Hybrid combination 2 (ICPH 2740) | | | |
| 1 | ICPA2047=ICPR2740<ICPH2740 | 0 | Over dominance |
| 2 | ICPA2047<ICPR2740<ICPH2740 | 1477 (11.76 %) | +ve over dominance |
| 3 | ICPA2047<ICPH2740=ICPR2740 | 3 (0.02 %) | Dominance |
| 4 | ICPA2047<ICPH2740<ICPR2740 | 5319 (42.36 %) | Partial dominance |
| 5 | ICPA2047=ICPH2740<ICPR2740 | 0 | -ve dominance |
| 6 | ICPH2740<IPCA2047<ICPR2740 | 7 (0.06 %) | -ve over dominance |
| 7 | ICPH2740<IPCA2047=ICPR2740 | 0 | Dominance |
| 8 | ICPH2740<ICPR2740< ICPA2047 | 2344 (18.67 %) | -ve over dominance |
| 9 | ICPH2740=ICPR2740< ICPA2047 | 1 (0.01 %) | -ve dominance |
| 10 | ICPR2740<ICPH2740< ICPA2047 | 2746 (21.87 %) | Partial dominance |
| 11 | ICPR2740< ICPA2047=ICPH2740 | 0 | Dominance |
| 12 | ICPR2740< ICPA2047<ICPH2740 | 42 (0.33 %) | +ve over dominance |
| 13 | ((IPCA2047+ICPR2740)/2) =ICPH2740 | 619 (4.93 %) | Additive |
| Total | | 12558 |  |

**Table S19.** Identification of DEGs associated DMRs present in genic and flanking (2 kb up and downstream) regions

| Combinations | Total  DEGs (FDR <0.001 and Fold Change >1) | DEGs-gene and flanking DMRs | Percent DEGs-gene and flanking DMRs | Up-regulated genes | Down-regulated genes | P-value between fold enrichment of up-and down-regulated genes |
| --- | --- | --- | --- | --- | --- | --- |
| *ICPH 2671 and its parental lines* | | | | | |  |
| ICPA 2043/ICPR 2671 | 7185 | 1162 | 15.4 | 862 | 300 | 1.2882E-270 |
| ICPA 2043/ICPH 2671 | 378 | 46 | 12.2 | 33 | 13 | 1.78E-22 |
| ICPR 2671/ICPH 2671 | 6022 | 765 | 12.7 | 235 | 530 | 5.9E-230 |
| *ICPH 2740 and its parental lines* | | | | | |  |
| ICPA 2047/ICPR 2740 | 23956 | 3521 | 14.69 | 2553 | 968 | 0.00124526 |
| ICPA 2047/ICPH 2740 | 15434 | 1826 | 11.15 | 1641 | 185 | 1.6E-120 |
| ICPR 2740/ICPH 2740 | 144 | 12 | 8.333 | 12 | 0 |  |

**Table S20.** REVIGO amalgamated GO biological process associated with the hybrid-MPV DEGs for ICPH 2671 and ICPH 2740 hybrid combinations. The single unified GO terms obtained from REVIGO are clustered according to a broader classification. To further simplify the interpretation of the findings, the clusters are then amalgamated to produce 'major clusters' with the broad classification (*Dataset*)
